# Compositional and provenance study of glass beads from archaeological sites in Mali and Senegal at the time of the first Sahelian states

**DOI:** 10.1371/journal.pone.0242027

**Published:** 2020-12-02

**Authors:** Miriam Truffa Giachet, Bernard Gratuze, Anne Mayor, Eric Huysecom

**Affiliations:** 1 Anthropology Unit, Department of Genetics and Evolution, Laboratoire Archéologie et Peuplement de l’Afrique (APA), University of Geneva, Geneva, Switzerland; 2 Centre Ernest-Babelon de l’Institut de recherche sur les archéomatériaux (IRAMAT-CEB, UMR 5060), CNRS/University of Orléans, Orléans, France; 3 Global studies Institute, University of Geneva, Geneva, Switzerland; Universita degli Studi di Milano, ITALY

## Abstract

The presence of glass beads in West African archaeological sites provides important evidence of long-distance trade between this part of the continent and the rest of the world. Until recently, most of these items came from historical Sub-Saharan urban centers, well known for their role in the medieval trans-Saharan trade. We present here the chemical analysis by Laser Ablation-Inductively Coupled Plasma-Mass Spectrometry (LA-ICP-MS) of 16 glass beads found in three rural sites excavated during the past decade: the funerary site of Dourou-Boro and settlement sites of Sadia, in central Mali, as well as the settlement site of Djoutoubaya, in eastern Senegal, in contexts dated between the 7^th^-9^th^ and the 11^th^-13^th^ centuries CE. Results show that the raw materials used to manufacture the majority of the glass most probably originated in Egypt, the Levantine coast and the Middle East. One bead is of uncertain provenance and shows similarities with glass found in the Iberian Peninsula and in South Africa. One bead fragment found inside a tomb is a modern production, probably linked to recent plundering. All of these ancient beads were exchanged along the trans-Saharan trade routes active during the rise of the first Sahelian states, such as the Ghana and the Gao kingdoms, and show strong similarities with the other West African bead assemblages that have been analysed. Despite the remoteness of their location in the Dogon Country and in the Falémé River valley, the beads studied were therefore included in the long-distance trade network, via contacts with the urban commercial centers located at the edge of the Sahara along the Niger River and in current southern Mauretania. These results bring a new light on the relationships between international and regional trade in Africa and highlight the complementarity between centres of political and economic power and their peripheries, important because of resources like gold for eastern Senegal.

## Introduction

### Scope and objectives

Almost every society is or has been involved in the use of beads made from a great variety of materials. These objects are interesting as they can provide insightful information about these societies, from their cultural contexts to the political and social customs, and from their economic system to the technological questions linked to bead production [1].

Glass was invented almost four millennia ago and the production technologies and raw materials used for glassmaking differ throughout history. Beads are among the first objects produced with this synthetic material and the chemical analysis of archaeological glass beads is useful for identifying provenance and ancient trade routes [2, 3].

Presented in this article is the techno-stylistic analysis of 21 glass beads and the chemical analysis by LA-ICP-MS of a subgroup of 16 glass beads found in three West African archaeological sites in contexts dating between the 7^th^-9^th^ and the 11^th^-13^th^ centuries CE. The sites of Dourou-Boro and Sadia, in Central Mali, and Djoutoubaya, in Eastern Senegal, were excavated between 2007 and 2018 by the research team of the University of Geneva’s laboratory *Archéologie et Peuplement de l’Afrique* (APA), within the research project Human Population and Palaeoenvironment in Africa (HPPA) led by professor Éric Huysecom. The aim of this work is to identify the origin of the glass beads analysed and to evaluate the implications in relation to the trade of these items in West Africa at the time of the first Sahelian states. A particularly interesting research question concerns the integration of the hinterland with the trans-Saharan trade networks and the evaluation of the connection between the urban centres directly involved in the long-distance exchanges, on which the research has been mainly focusing on, and the peripheral rural areas.

### Background: The production and trade of glass

In order to understand the provenance and trade of glass beads, it is essential to differentiate between primary production centres, where raw glass was produced from raw materials, and secondary working sites, where glass objects were manufactured. Especially in ancient times, the glassworking centres were numerous and disseminated, whereas the glassmaking sites were limited in number and generally located in proximity of the raw material sources [2–4]. Through the chemical analysis of glass, it is possible to identify the probable area of origin of the raw materials used, but it is very difficult to locate the beadmaking sites, except for very specific bead types, diagnostic of a particular workshop. Archaeometric studies of archaeological glass dating between the 1^st^ millennium BCE and the 1^st^ millennium CE have steadily increased in the recent decades, but the research has been mainly focused on the Eastern Mediterranean basin and in Western Asia. Current models of glass production and distribution might therefore be implicitly affected by this preferential focal point and more systematic studies need to be directed towards other geographical areas in order to avoid a biased picture.

Between the 8^th^ and the 15^th^ centuries CE the active glassmaking centres were mainly located in the Mediterranean basin and the Middle East (mostly producing plant ash glass), in the south and south-east of Asia and in the Far East (mostly producing mineral soda and plant ash glass, as well as lead and lead-barium glass), and in continental Europe and Italy (producing respectively potash-lime glass from wood ash and soda-lime glass from coastal plant ash) [3]. As for the African continent, the only primary glassmaking centres south of the Sahara were located at Ile-Ife, in southern Nigeria, dating between the 9^th^ and the 15^th^ centuries CE. The glass produced there had a very specific composition, *i*.*e*. high lime high alumina glass (HLHA), which is not produced anywhere else in the world [5–8]. In sub-Saharan Africa, glass beads were therefore mostly exotic items, traded in various quantities through diverse long-distance exchange routes.

The archaeological research of the last two decades has shown that the first contacts and exchanges of goods between North and West Africa through the Sahara desert were active as early as the first millennium BCE, with the Garamantes of Fezzan playing a crucial role in the trade [9–15]. During this period, however, the exchanges were quite scattered, as shown by the limited archaeological evidence. Nonetheless, some glass beads were found in archaeological contexts dating from before the Arabic conquest of North Africa and located mainly along the Niger River [16]. After the 7^th^ century CE, the amount of goods and the complexity of the commercial system increased exponentially, flourishing particularly during the expansion of the Sahelian states between the 10^th^ and the 15^th^ centuries CE [17, 18]. In the context of long-, medium-, and short-distance trade, the main caravan towns active during the development of the West African kingdoms were located in the Sahelian Strip, directly connected to the trade routes and the Arab-Berber merchants, *e*.*g*. Tegdaoust and Koumbi Saleh in southern Mauritania, within the context of the Ghana kingdom, or Gao, Timbuktu, and Essouk in northern Mali, under the authority of the Gao kingdom. From these caravan towns, the goods were dispatched at a regional level to the main market towns, such as for example Jenne-Jeno in Mali, and from there onto the local markets and villages [19]. Glass beads acquired during this period had an important economic and social value, and were frequently mentioned in the Arabic texts in relation to trans-Saharan trade, *e*.*g*. the travel accounts written by al-Zuhri, al-Idrisi, al-Qazwīnī, Ibn Battuta, and Yuqut [20–24]. Importantly, glass beads have been found in varying amounts in many West African archaeological contexts dating between the second half of the 1^st^ and the first half of the 2^nd^ millennium CE, the quantities depending on the function and location of the site. The largest collections are generally found in the sites acting as seats of political and economic power, such as Igbo-Ukwu in Nigeria [25, 26] or Gao in Mali [27]. On the contrary, until now, very few imported glass beads have been found in rural peripheral sites and analysed. Moreover, during this time, centres for reworking the imported glass emerged in order to meet the demand and the local taste, as in Gao Saney in Mali [24, 28]. A review of the main sites delivering glass beads in West Africa can be found in a very recent paper concerning the glass beads found in Gao [27]. The map presented in the article shows how the sites are mainly located at the southern fringe of the Sahara, as well as along the Niger River, where research has been mainly focusing. Thus, it is important to keep in mind that, with a few exceptions (e.g. Kissi, in Burkina Faso), the compositional analysis of glass beads has been mostly performed on assemblages found in major urban centres with direct connections to the trans-Saharan trade.

Trans-Saharan trade began to decline in the 15^th^ century CE with the arrival of the Europeans along the African coasts and increased exchanges by way of the Atlantic Ocean.

## Materials and methods

For Mali, permission for archaeological research was granted by the CNRST (centre national de la recherche scientifique et technique), and temporal export for analysis was granted by ISH (Institut des sciences humaines). For Senegal, permissions for archaeological research and temporal export for analysis were granted by IFAN (Institut fondamental d'Afrique noire).

### Archaeological contexts and glass bead assemblages

#### Dourou-Boro, Mali

**Fig 1:**
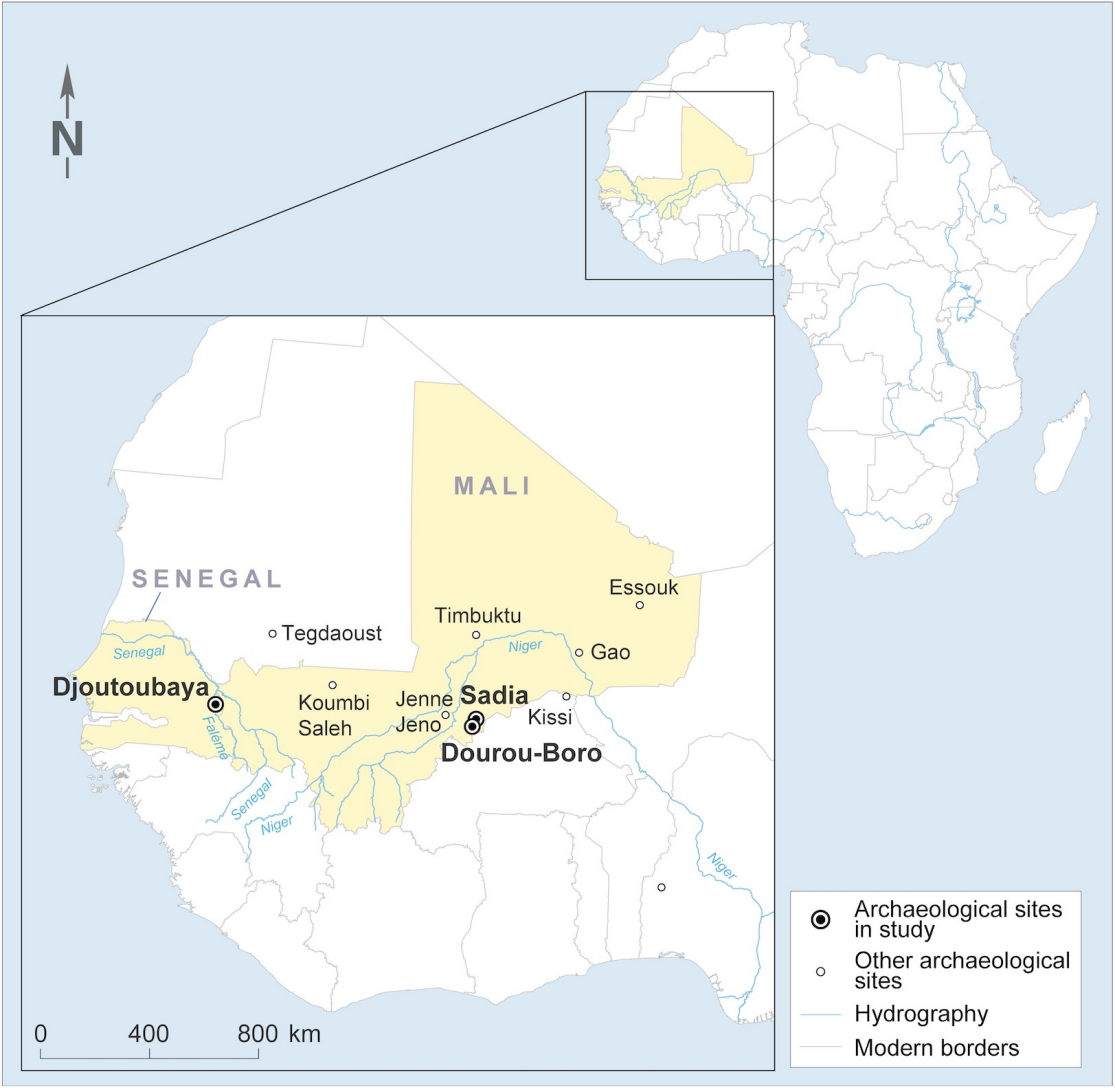
Archaeological sites in West Africa. Location of the archaeological sites where the analysed glass beads were found, as well as of other West African archaeological sites mentioned in this paper (map: courtesy of CIA’s *The World Factbook* 2020, modified).

**Fig 2:**
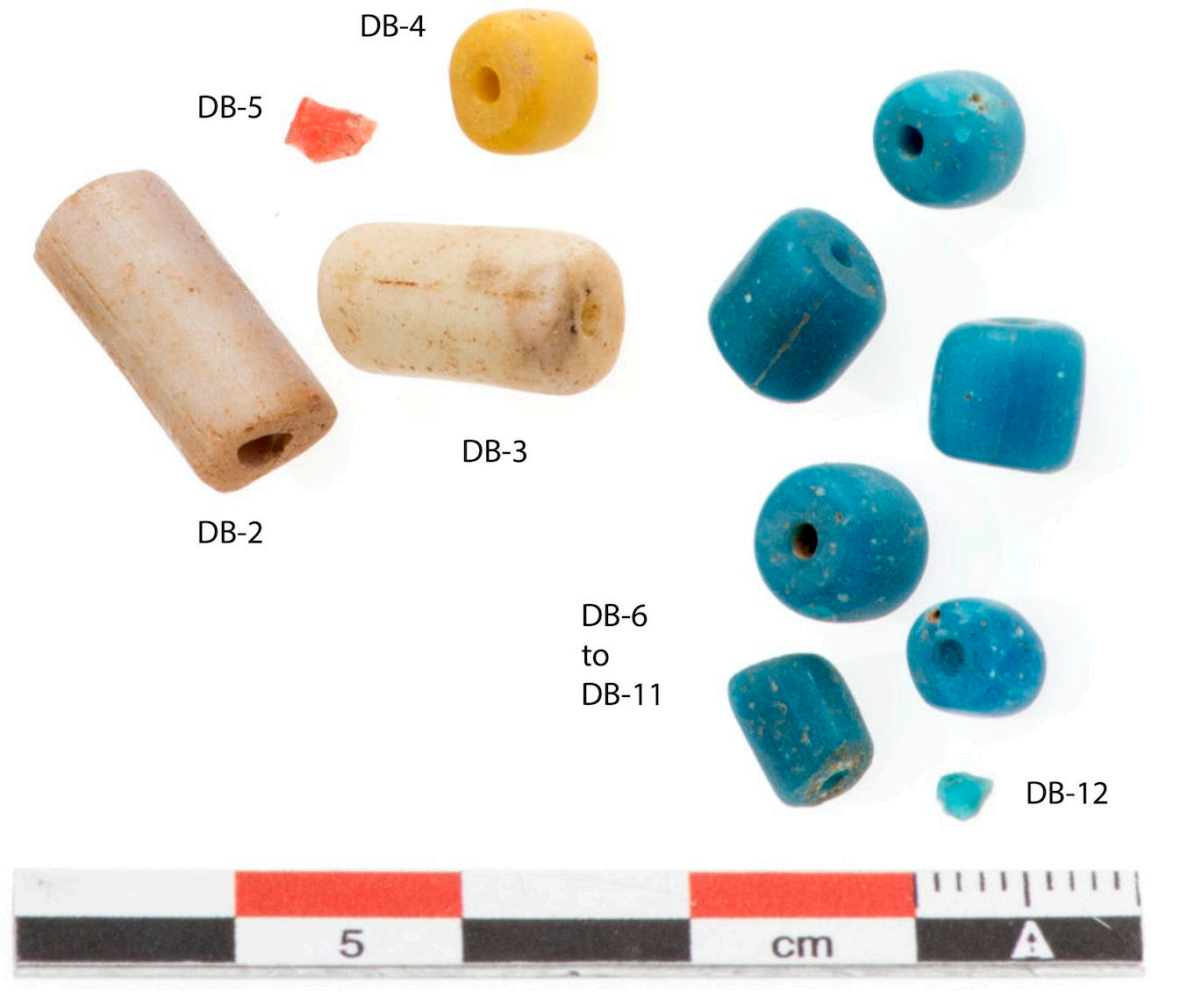
Glass beads found in Dourou-Boro, Mali.

Dourou-Boro is a funerary rock shelter situated in the Dogon Country in Central Mali, specifically in the Bandiagara Escarpment (Fig 1). This archaeological site, excavated in 2007, is composed of several circular structures principally built from coiled and modelled clay, and decorated with finger impressions. These buildings used as collective graves show varying stages of preservation [29]. Most of the archaeological material was found on the bedrock in front of the tombs. This location is probably due to intentional ritual deposits combined with weathering and burrowing animal activities, which created cavities at the base of the structures causing the objects to roll outside. Among this material, consisting mainly of pottery sherds and numerous iron objects, were 11 glass beads, 7 carnelian beads, a quartz crystal, and a fragment of blue glass. In addition, a fragment of red glass was found inside one of the tombs, together with human bones and the remains of a wooden headrest. Radiocarbon AMS dating of 7 vegetal samples from the clay walls, 3 bone samples, and 1 wooden sample places the construction phase of the structures between 420 and 570 CE and the last funerary use of the site between 600 and 850 CE [29]. The assemblage of glass beads and fragments examined in this article (Fig 2) belong to this final phase of occupation.

Table 1 lists the morphological and visual characteristics of the glass samples found. Two turquoise beads (not displayed in Fig 2 and Table 1) were previously analysed by Dr Sonja Magnavita at the Goethe University of Frankfurt by means of EPMA and LA-ICP-MS [29] and are included in the data analysis.

**Table 1:** Morphological and visual characteristics of the 21 glass beads found in Dourou-Boro and Sadia (Mali), and in Djoutoubaya (Senegal).

Site	Sample	Length [mm]	Diameter [mm]	Longitudinal section	Cross section	Colour	Diaphaneity	Manufacture technique	Secondary modification
Dourou-Boro	**DB-2**	15.5	7.5	Cylindrical	Circular	PU: 7.5YR 4/4	Opaque	Drawn	None
	**DB-3**	13.8	7.4	Cylindrical	Circular	BE: 5.0Y 9/2	Opaque	Drawn	None
	**DB-4**	5.4	6.7	Cylindrical	Circular	Y: 2.5Y 6/8	Opaque	Drawn	Heat-rounded
	**DB-5**	n.a.	n.a.	n.a.	n.a.	R: 8.75R 4/14	Opaque	n.a.	n.a.
	**DB-6**	7.5	7.4	Cylindrical	Circular	T: 5.0B 4/6	Opaque	Drawn	Heat-rounded
	**DB-7**	6.9	6.7	Cylindrical	Circular	T: 5.0B 4/6	Opaque	Drawn	Heat-rounded
	**DB-8**	5.8	7.2	Cylindrical	Circular	T: 5.0B 4/6	Opaque	Drawn	Heat-rounded
	**DB-9**	6	6.4	Cylindrical	Circular	T: 5.0B 4/6	Opaque	Drawn	Heat-rounded
	**DB-10**	6.7	6.2	Cylindrical	Elliptical	T: 5.0B 4/6	Opaque	Drawn	Heat-rounded
	**DB-11**	4.2	6.7	Cylindrical	Elliptical	T: 5.0B 4/6	Opaque	Drawn	Heat-rounded
	**DB-12**	n.a.	n.a.	n.a.	n.a.	T: 2.5B 6/7	Opaque	n.a.	n.a.
Djoutoubaya	**DJ16-3**	3.5	5.7	Short barrel	Elliptical	B: 5.0PB 3/4	Translucent	Drawn	Heat-rounded
	**DJ16-5t**	1.6	3.1	Cylindrical	Circular	T: 10.0BG 7/4	Translucent	Drawn	None
	DJ16-9*	8.2	14.8	Short barrel	Circular	n.a.	n.a.	Drawn?	Heat-rounded?
	DJ16-11*	2.7	4.5	Cylindrical	Elliptical†	n.a.	n.a.	n.a.	n.a.
	**DJ16-12**	5.9	n.a.	Cylindrical	n.a.	T: 5.0B 5/7	Translucent	Drawn	n.a.
	**DJ17-5**	7.9	6.3	Cylindrical†	Elliptical	T: 10.0B 4/10	Translucent	Drawn	None
	DJ17-6*	2.9	6.2	Short barrel	Elliptical	B: 5.0PB 3/4	Translucent	Drawn	Heat-rounded
Sadia	**SA11-2**	5	6.6	Cylindrical	Circular	G: 2.5G 6/4	Opaque	Drawn	Heat-rounded, ends ground flat
	SA11-5*	2.6	7.3	Cylindrical	Circular	n.a.	Opaque?	Drawn	n.a.
	SA11-12*	2.4	7.1	Cylindrical	Circular	n.a.	Opaque?	Drawn	n.a.

*Beads not subjected to chemical analysis.

†Irregular shape.

Maximum length and diameter are given in millimetres. n.a. = not available. Munsell colour codes [30] are indicated for each of the following colours: B = blue, BE = beige, G = green, PU = purple, R = red, T = turquoise, Y = yellow.

#### Sadia, Mali

**Fig 3:**
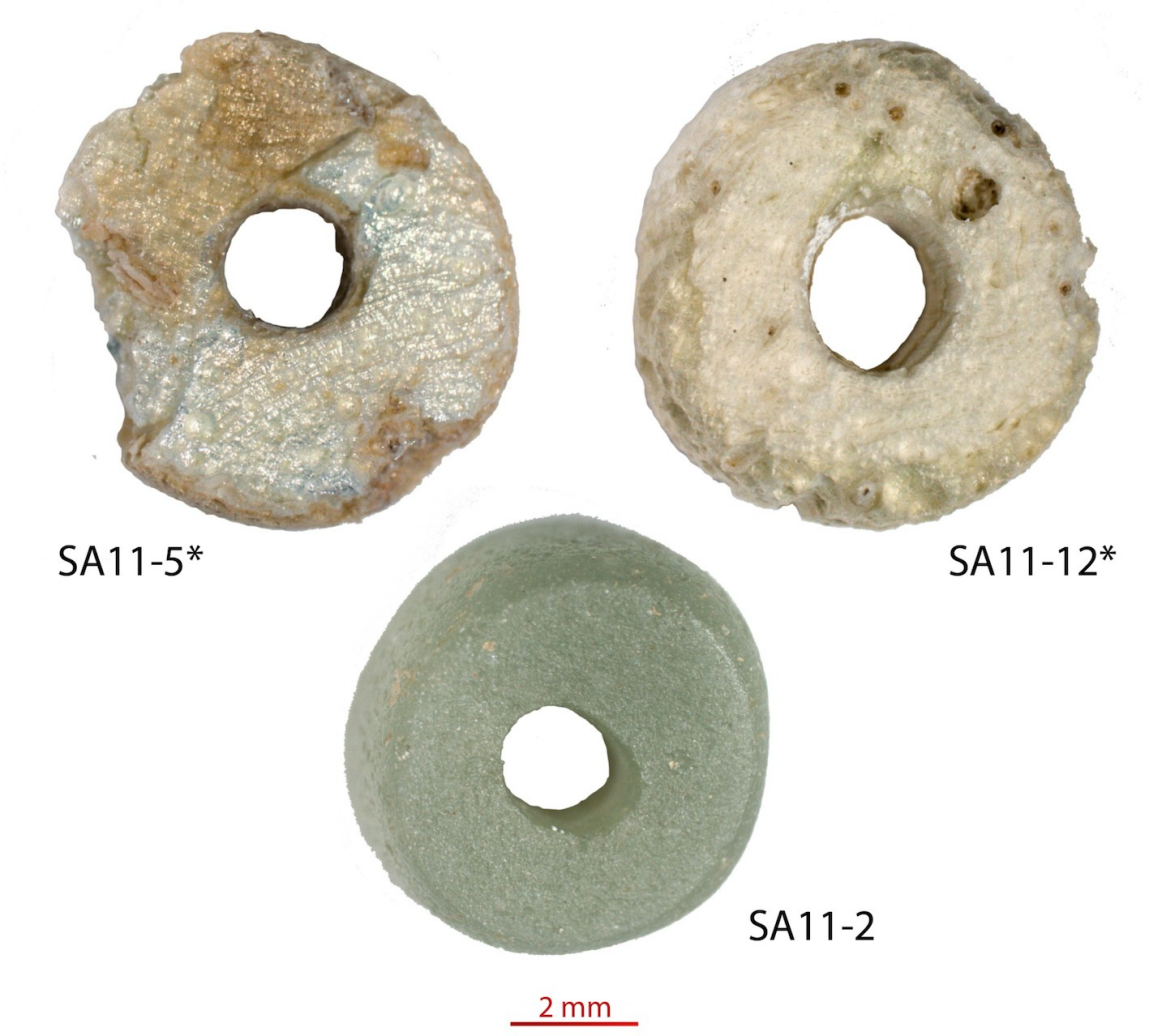
Glass beads found in Sadia, Mali. The two deteriorated beads not subjected to chemical analysis are indicated with “*”.

Sadia is a settlement site located in the Seno-Gondo plain in the Guringin valley, at about 15 km from the Bandiagara Escarpment in Mali (Fig 1). The site comprises 5 settlement mounds (tells) excavated in 2010 and 2011. According to the radiocarbon dates of 27 charcoal samples, the stratigraphic sequence is composed by one pre-tell phase dated before the 1^st^-3^rd^ c. CE, and three phases for the tell occupation, between the mid 8^th^ and the late 13^th^ centuries CE [31]. The extensive excavation of the site delivered a large quantity of potsherds and metallic objects, in addition to various grinding tools, clay objects, stone beads, metallurgical waste, as well as 3 glass beads (Fig 3). These objects were found in contexts dating between the 11^th^ and the 13^th^ centuries CE.

Two of these beads are in an advanced stage of degradation and therefore were not therefore chemically analysed. The morphological and visual characteristics of the glass beads are listed in Table 1.

#### Djoutoubaya, Senegal

**Fig 4:**
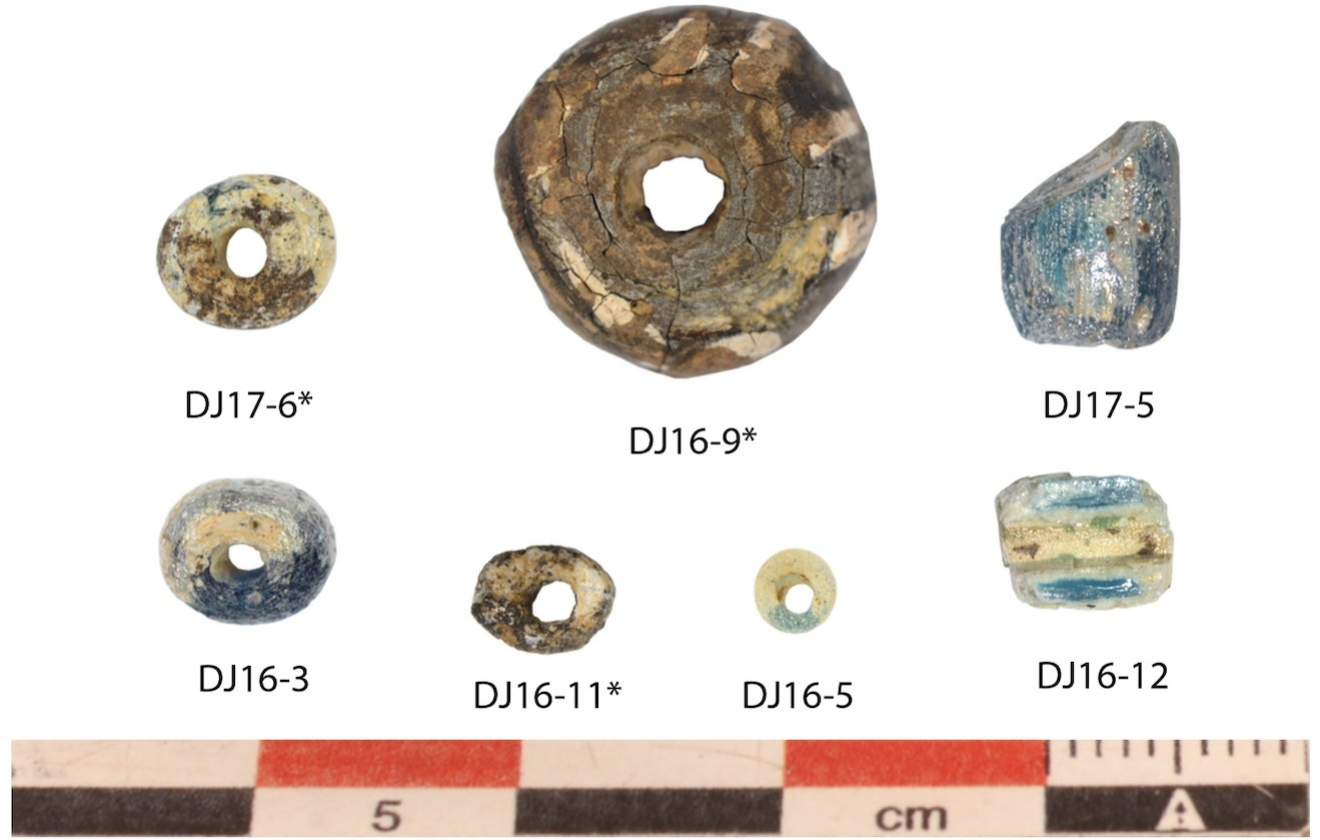
Glass beads found in Djoutoubaya, Senegal. The three deteriorated beads not subjected to chemical analysis are indicated with “*”.

Djoutoubaya is a settlement site located on the right bank of the Falémé River in Senegal (Fig 1). Four excavation campaigns have taken place since 2014 and have revealed the base of a quadrangular building composed by rectangular mud bricks, which is a fairly unusual shape and technique for this area during the “medieval” era [32]. The excavations showed a complex stratigraphic sequence and the radiocarbon dates from numerous charcoal samples identified of 4 occupational phases between the 9^th^-10^th^ and the 13^th^-14^th^ centuries CE, the building being used between the 12^th^ and the 13^th^ centuries CE [32]. In addition, the discovery of several fragments and 2 complete small clay pots thought to be crucibles for gold metallurgy opens up to different interpretations about the function of the site [33, 34]. Djoutoubaya is centrally located in one of the gold production areas active during the Ghana and Mali empires. Among the archaeological materials unearthed, such as potsherds, metallic objects, cowrie shells, spindle whorls, as well as stone, ivory, metal and clay beads, were 7 glass beads (Fig 4), whose characteristics are listed in Table 1. The glass beads were found in different sectors of the site, in contexts dating between the 10^th^ and the 13^th^ centuries CE. Three of the glass beads show a severe degradation of the glass matrix and therefore were not chemically analysed.

### Analytical methods

The 16 non-degraded archaeological glass beads were analysed by Laser Ablation–Inductively Coupled Plasma–Mass Spectrometry (LA–ICP–MS) at the *Institut de Recherche sur les Archéomatériaux* (CEB-IRAMAT, CNRS/Orleans University, France). The instrumentation used consists of a Resonetics M50E excimer laser working at 193 nm and a Thermo Fisher Scientific ELEMENT XR mass spectrometer. The samples did not receive any specific preparation and were analysed within the standard Resonetic S155 cell. The pulsed laser beam was operating at 5 mJ energy and 10 Hz pulse frequency. The beam diameter was adjusted between 50 and 100 μm in order to minimise the signal saturation of some elements, leaving craters 150–250 μm deep. The standard analytical protocol includes a 20 s pre-ablation followed by 30 s signal acquisition, which corresponds to 10 mass scans but for the samples presenting a thick alteration layer on surface, the pre-ablation time was increased in order to have a reliable signal from the unaltered bulk glass. The sampled aerosol is carried to the injector inlet of the plasma torch by an argon/helium flow (at a rate of 1 L/min for Ar and 0.65 L/min for He) where it is dissociated, atomised, and ionised. The signal in count-per-second was measured in low-resolution mode for 58 isotopes, from lithium to uranium. This analytical protocol allows for the determination of nearly all the elements present in ancient glass with the exception of sulphur. External calibration was performed using the Standard Reference Materials from the National Institute of Standards and Technology (NIST SRM) 610, the Corning reference glasses B, C, and D, as well as the in-house archaeological sample APL1 used for chlorine quantification; reference materials Corning A and NIST SRM612 were analysed with the beads as unknown samples. ^28^Si was used as an internal standard. The concentrations of each element were calculated according to the protocol detailed in [35] and [36], thus obtaining detection limits from 0.01% to 0.1% for major elements, and from 20 to 500 ppb for minor and trace elements.

## Results

Table 2 shows the reduced compositions of the 16 monochrome glass beads, as well as of the two glass beads from Dourou-Boro previously analysed [29] (cf. *supra*). The reduced composition has been calculated following the method of Brill [37], excluding iron and including chlorine and phosphorus pentoxide, in order to better reflect the base composition of the glasses.

**Table 2:** Reduced composition (*) of the analysed glass beads by site.

Sample	Na_2_O*	MgO*	Al_2_O_3_*	SiO_2_*	P_2_O_5_*	Cl*	K_2_O*	CaO*
**DB-2pu**	15.7%	1.03%	1.58%	73.0%	0.36%	1.40%	2.64%	4.21%
**DB-3be**	17.7%	1.65%	2.01%	69.6%	0.34%	1.43%	2.37%	4.91%
**DB-4y**	13.6%	4.09%	3.27%	68.2%	0.31%	0.74%	2.92%	6.91%
**DB-5r**	15.5%	0.05%	14.73%	65.4%	0.13%	0.10%	2.51%	1.64%
**DB-6t**	15.5%	2.37%	1.53%	70.4%	0.61%	1.27%	2.99%	5.36%
**DB-7t**	17.4%	2.57%	1.60%	69.4%	0.32%	1.42%	2.10%	5.20%
**DB-8t**	18.6%	1.85%	1.81%	68.4%	0.42%	1.56%	2.01%	5.41%
**DB-9t**	16.1%	4.72%	1.46%	66.9%	0.28%	0.97%	3.24%	6.39%
**DB-10t**	16.8%	2.41%	1.79%	69.3%	1.00%	1.30%	1.66%	5.69%
**DB-11t**	16.7%	5.89%	0.57%	66.2%	0.15%	0.89%	3.18%	6.43%
**DB-12t**	13.2%	0.85%	1.03%	76.3%	0.39%	1.42%	3.38%	3.49%
**DB-SM1t**^**§**^	15.3%	1.45%	1.45%	69.8%	0.55%	n.a.	3.53%	7.86%
**DB-SM2t**^**§**^	17.0%	2.49%	1.58%	70.6%	0.42%	n.a.	2.65%	5.25%
**DJ16-3b**	14.2%	4.35%	2.92%	68.6%	0.20%	0.59%	2.95%	6.22%
**DJ16-5t**	12.8%	2.94%	3.49%	73.2%	0.52%	0.91%	1.21%	4.87%
**DJ16-12t**	19.4%	2.73%	1.66%	66.1%	0.52%	1.70%	2.51%	5.34%
**DJ17-5t**	16.8%	1.61%	1.52%	72.1%	0.33%	1.36%	1.97%	4.28%
**SA11-2g**	16.5%	4.61%	6.73%	61.1%	0.41%	0.72%	3.06%	6.87%

§Dourou-Boro samples previously analysed.

The last letters of the sample names indicates the colour of the glass (cf. Table 1 for abbreviations). Concentrations are given in wt%, the total being 100 wt%. n.a.: not available.

Two principal glass compositions were identified, namely soda-lime-silica glass (17 samples) and soda-alumina-silica glass with a high boron content (1 sample, DB-5r, B_2_O_3_: 3.4%). The samples of the main group show a content of SiO_2_* ranging from 61.1 to 76.3 wt%, Na_2_O* from 12.8 to 19.4 wt%, CaO* from 3.5 to 7.9 wt%, and Al_2_O_3_* from 0.6 to 6.7 wt%. These values indicate that the beads were made with a soda-lime-silica glass produced from more or less pure sands. Moreover, except for sample DB-12t, the strontium content of the glasses (251–1062 ppm) suggests a coastal origin for the sand, which is characterised by Sr > 300 ppm due to the presence of beach shells [38, 39]. Besides, samples DB-12t shows a strontium to calcium oxide ratio (Sr ppm/CaO wt% = 5569) in line with the other samples (Sr ppm/CaO wt% > 5000), suggesting that the same type of vitrifier, although of variable purity, was used (cf. *infra*). Concerning the flux, MgO* and K_2_O* concentrations range from 0.9 to 5.9 wt% and from 1.2 and 3.5 wt% respectively, their total being always higher than 3.6 wt%, indicating therefore that soda plant ash was probably used to lower the melting temperature of the silica. This is confirmed by the high P_2_O_5_* level (0.15–1.0 wt%), typical of this kind of flux [3].

**Fig 5:**
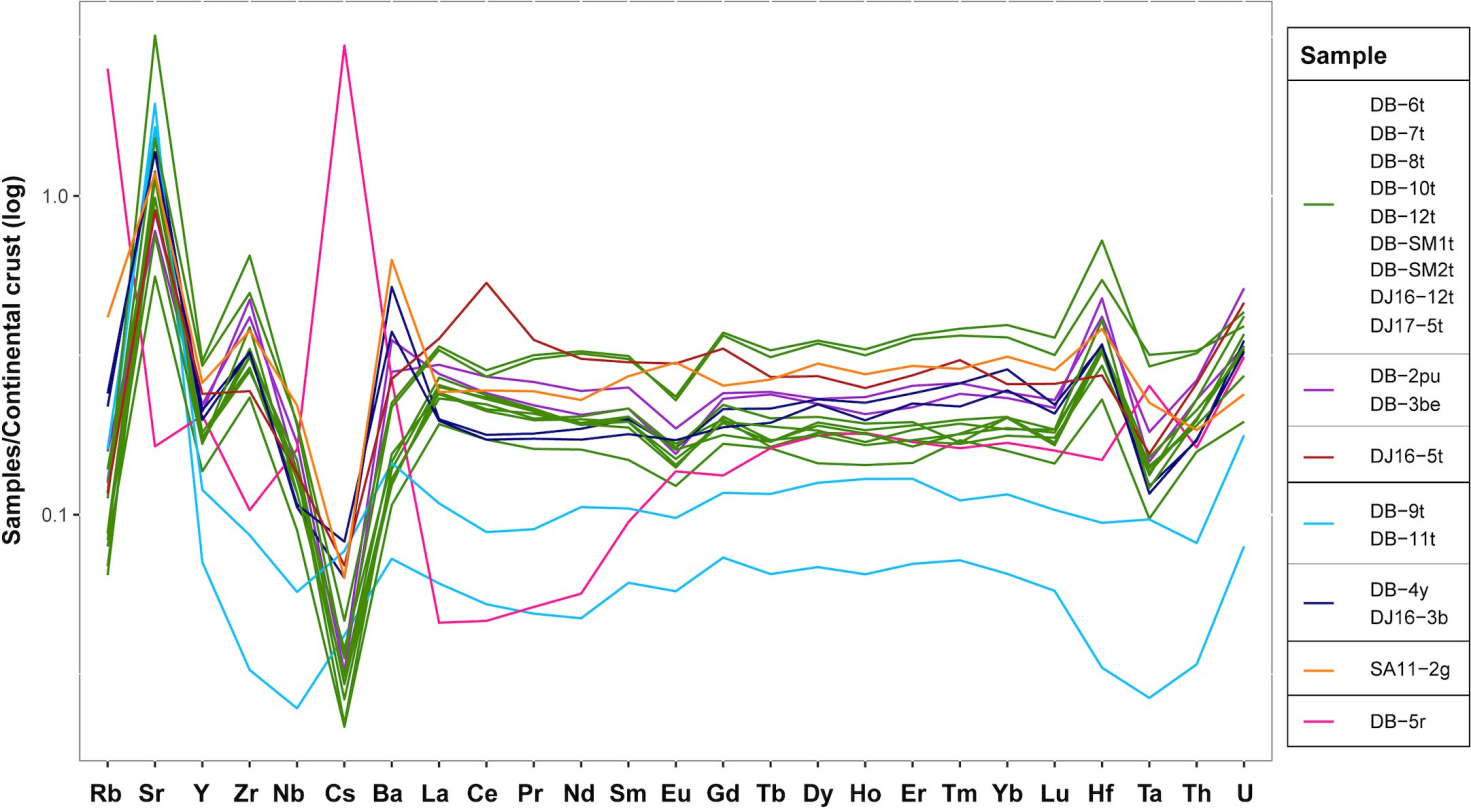
Sample grouping based on trace element content. Trace elements trends from the analysed samples revealing various subgroups within the two main clusters.

Based on the alumina, magnesia and lime content, three subgroups were identified for the soda-lime-silica glass samples, namely soda-lime-silica glass with low lime and magnesia (12 samples), soda-lime-silica glass with high magnesia (4 samples), and soda-lime-silica glass with high alumina (1 sample). Trace element analysis confirms this subdivision and additionally reveals various subgroups within the two main clusters (Fig 5).

The full chemical composition of the samples, grouped into the chemical glass types identified, is presented in Table 3. The results are given for all elements having significant levels; the major and minor elements concentrations are given in weight percent (wt%) and the trace elements as part per millions (ppm).

**Table 3:** Chemical composition of the glass beads from Dourou-Boro, Djoutoubaya, and Sadia (cf. Table 1 for abbreviations).

Sample	Na_2_O	MgO	Al_2_O_3_	SiO_2_	P_2_O_5_	Cl%	K_2_O	CaO	TiO_2_	MnO	Fe_2_O_3_	CuO	As_2_O_3_	SnO_2_	Sb_2_O_3_	PbO
*Soda-lime-silica glass with low lime and magnesia*																
**DB-6t**	14.9%	2.29%	1.48%	68.0%	0.59%	1.23%	2.89%	5.18%	0.12%	0.017%	0.58%	1.67%	0.0038%	0.24%	0.0004%	0.64%
**DB-7t**	16.7%	2.48%	1.54%	66.9%	0.31%	1.37%	2.02%	5.01%	0.12%	0.016%	0.55%	2.18%	0.0058%	0.16%	0.0004%	0.46%
**DB-8t**	17.6%	1.75%	1.71%	64.7%	0.40%	1.47%	1.90%	5.12%	0.14%	0.024%	0.69%	1.95%	0.0053%	0.82%	0.0015%	1.61%
**DB-10t**	16.1%	2.31%	1.71%	66.4%	0.96%	1.25%	1.60%	5.45%	0.13%	0.018%	0.70%	2.19%	0.0070%	0.22%	0.0011%	0.75%
**DB-12t**	12.6%	0.81%	0.98%	72.8%	0.37%	1.36%	3.22%	3.34%	0.086%	0.016%	0.42%	1.56%	0.016%	1.05%	0.0006%	1.29%
**DJ16-12t**	18.5%	2.61%	1.58%	63.2%	0.50%	1.63%	2.40%	5.11%	0.12%	0.038%	0.62%	2.03%	0.0046%	0.39%	0.0005%	1.07%
**DJ17-5t**	16.2%	1.55%	1.46%	69.2%	0.31%	1.31%	1.89%	4.10%	0.11%	0.065%	0.65%	2.39%	0.0056%	0.24%	0.0008%	0.47%
**DB-SM1t**^**§**^	15.2%	1.43%	1.43%	69.0%	0.54%	n.a.	3.49%	7.77%	0.20%	0.23%	0.79%	2.05%	n.a.	n.a.	n.a.	2.16%
**DB-SM2t**^**§**^	16.9%	2.46%	1.56%	69.9%	0.42%	n.a.	2.62%	5.20%	0.19%	0.21%	0.69%	2.36%	n.a.	n.a.	n.a.	1.48%
**DB-2pu**	15.0%	0.99%	1.51%	69.7%	0.34%	1.34%	2.52%	4.02%	0.13%	0.59%	0.60%	0.023%	0.0031%	0.91%	0.0004%	2.16%
**DB-3be**	16.9%	1.57%	1.92%	66.2%	0.32%	1.36%	2.25%	4.67%	0.16%	0.36%	0.81%	0.030%	0.0028%	0.87%	0.0007%	2.47%
**DJ16-5t**	12.5%	2.86%	3.40%	71.2%	0.50%	0.89%	1.18%	4.73%	0.16%	0.12%	1.11%	0.93%	0.016%	0.024%	0.0047%	0.13%
*Soda-lime-silica glass with high magnesia*																
**DB-9t**	15.3%	4.51%	1.39%	63.9%	0.27%	0.93%	3.09%	6.10%	0.062%	0.073%	0.61%	1.07%	0.017%	0.75%	0.0009%	1.54%
**DB-11t**	16.1%	5.69%	0.55%	63.9%	0.14%	0.86%	3.07%	6.21%	0.031%	0.024%	0.25%	1.35%	0.011%	0.57%	0.0009%	0.82%
**DB-4y**	12.8%	3.85%	3.07%	64.1%	0.29%	0.70%	2.75%	6.49%	0.11%	1.70%	0.77%	0.029%	0.017%	0.50%	0.0074%	2.67%
**DJ16-3b**	13.7%	4.19%	2.81%	66.0%	0.19%	0.57%	2.84%	5.98%	0.11%	0.53%	1.36%	0.25%	0.010%	0.19%	0.0002%	0.94%
*Soda-lime-silica glass with high alumina*																
**SA11-2g**	15.8%	4.43%	6.47%	58.7%	0.40%	0.69%	2.94%	6.60%	0.22%	0.065%	1.47%	0.66%	0.0043%	0.41%	0.0034%	0.97%
*Soda-alumina-silica glass with high boron*																
**DB-5r**	14.8%	0.05%	14.1%	62.6%	0.13%	0.10%	2.40%	1.57%	0.037%	0.0037%	0.11%	0.0005%	0.0020%	0.0001%	0.0002%	0.0026%
**Sample**	**Li**	**B**	**Ti**	**V**	**Cr**	**Mn**	**Co**	**Ni**	**Cu**	**Zn**	**Ga**	**As**	**Se**	**Rb**	**Sr**	**Y**
*Soda-lime-silica glass with low lime and magnesia*																
**DB-6t**	73.5	123	694	9.12	10.3	132	1.42	9.09	13312	75.1	2.31	29.0	n.d.	10.8	329	4.02
**DB-7t**	43.4	78.1	712	10.6	9.08	124	1.51	8.31	17376	38.6	2.56	44.0	n.d.	6.86	398	4.08
**DB-8t**	38.3	85.9	830	10.0	11.26	184	2.00	9.49	15603	77.2	2.79	39.8	n.d.	5.37	329	4.06
**DB-10t**	70.7	93.0	773	10.4	11.58	138	1.70	10.4	17523	53.9	2.60	52.8	n.d.	5.04	393	4.20
**DB-12t**	16.8	52.1	514	6.20	6.59	125	1.52	7.49	12472	63.5	1.62	119	n.d.	6.20	186	3.28
**DJ16-12t**	61.7	124	715	10.4	10.2	295	2.45	12.8	16226	205	2.71	34.9	n.d.	6.48	376	4.35
**DJ17-5t**	32.0	84.0	677	8.68	12.5	505	1.76	11.2	19074	49.2	2.52	42.6	n.d.	6.81	251	4.00
**DB-SM1t**^**§**^	42.8	83.5	1175	12.1	8.84	1778	4.64	16.9	16387	1332	6.38	n.a.	n.a.	9.11	1062	7.28
**DB-SM2t**^**§**^	74.8	103	1142	11.5	13.2	1615	3.06	12.9	18816	263	6.27	n.a.	n.a.	8.78	505	7.04
**DB-2pu**	23.7	63.6	803	10.6	10.7	4547	4.81	5.46	187	33.7	2.88	23.5	n.d.	12.3	259	5.05
**DB-3be**	43.1	87.3	945	13.9	10.9	2777	4.73	7.39	239	41.4	3.39	21.3	n.d.	9.82	296	5.31
**DJ16-5t**	38.3	110	937	15.5	20.8	953	32.1	38.5	7431	1501	5.17	124	n.d.	9.10	300	5.74
*Soda-lime-silica glass with high magnesia*																
**DB-9t**	23.7	116	374	11.0	26.3	563	8.92	50.6	8526	2032	1.74	131	n.d.	12.5	547	2.87
**DB-11t**	17.6	82.4	184	5.38	16.6	187	5.15	66.3	10799	2472	0.80	82.5	n.d.	9.97	649	1.70
**DB-4y**	13.5	140	681	16.2	48.9	13163	8.52	20.9	235	56.3	4.61	132	n.d.	17.0	506	5.11
**DJ16-3b**	14.6	128	678	15.2	47.5	4077	1052	377	2007	95.1	5.13	79.2	n.d.	18.7	457	4.76
*Soda-lime-silica glass with high alumina*																
**SA11-2g**	6.87	159	1312	20.2	25.8	500	8.52	17.6	5246	42.8	7.12	32.2	n.d.	32.4	392	6.22
*Soda-alumina-silica glass with high boron*																
**DB-5r**	3.86	10708	222	2.67	3.57	28.9	0.77	0.69	4.38	5.7	9.43	14.9	1814	196	55	4.91
**Sample**	**Ba**	**La**	**Ce**	**Pr**	**Nd**	**Sm**	**Eu**	**Gd**	**Tb**	**Dy**	**Ho**	**Er**	**Tm**	**Yb**	**Lu**	**Hf**
*Soda-lime-silica glass with low lime and magnesia*																
**DB-6t**	74.4	7.25	12.8	1.39	5.19	0.99	0.18	0.81	0.11	0.67	0.13	0.36	0.05	0.37	0.06	1.63
**DB-7t**	85.5	8.07	14.2	1.44	5.18	1.04	0.19	0.78	0.11	0.72	0.14	0.34	0.05	0.40	0.06	1.58
**DB-8t**	72.3	7.54	14.0	1.42	5.24	1.08	0.21	0.71	0.11	0.74	0.15	0.40	0.06	0.41	0.06	1.66
**DB-10t**	81.1	7.65	13.8	1.41	5.16	1.06	0.22	0.80	0.11	0.69	0.14	0.36	0.05	0.35	0.06	1.44
**DB-12t**	62.6	5.77	10.3	1.08	4.32	0.79	0.16	0.67	0.11	0.55	0.11	0.31	0.05	0.32	0.05	1.13
**DJ16-12t**	75.6	6.95	13.3	1.34	5.49	1.14	0.21	0.88	0.13	0.77	0.15	0.41	0.05	0.40	0.06	1.60
**DJ17-5t**	90.5	7.17	12.7	1.33	5.38	1.04	0.19	0.78	0.12	0.70	0.14	0.39	0.06	0.37	0.06	2.00
**DB-SM1t**^**§**^	127	9.85	16.3	2.06	8.64	1.63	0.31	1.49	0.21	1.34	n.a.	n.a.	n.a.	n.a.	n.a.	n.a.
**DB-SM2t**^**§**^	131	10.1	17.0	2.12	8.79	1.67	0.30	1.46	0.20	1.31	n.a.	n.a.	n.a.	n.a.	n.a.	n.a.
**DB-2pu**	206	8.30	14.4	1.48	5.55	1.14	0.20	0.92	0.15	0.85	0.17	0.46	0.07	0.46	0.08	2.34
**DB-3be**	164	8.86	16.3	1.75	6.60	1.33	0.24	0.96	0.16	0.88	0.19	0.53	0.08	0.48	0.08	2.05
**DJ16-5t**	156	10.7	32.0	2.37	8.33	1.59	0.39	1.33	0.18	1.03	0.20	0.57	0.09	0.51	0.09	1.34
*Soda-lime-silica glass with high magnesia*																
**DB-9t**	84.8	3.25	5.29	0.60	2.85	0.55	0.13	0.47	0.08	0.48	0.10	0.27	0.03	0.23	0.04	0.46
**DB-11t**	42.5	1.82	3.14	0.33	1.28	0.32	0.07	0.29	0.04	0.26	0.05	0.15	0.02	0.13	0.02	0.16
**DB-4y**	303	5.98	10.7	1.20	5.02	1.06	0.21	0.86	0.14	0.87	0.18	0.50	0.08	0.57	0.08	1.66
**DJ16-3b**	219	5.91	10.3	1.16	4.64	0.95	0.22	0.75	0.13	0.84	0.16	0.47	0.07	0.49	0.07	1.68
*Soda-lime-silica glass with high alumina*																
**SA11-2g**	368	7.27	14.7	1.63	6.18	1.44	0.39	1.02	0.17	1.13	0.22	0.62	0.09	0.63	0.10	1.87
*Soda-alumina-silica glass with high boron*																
**DB-5r**	154	1.37	2.78	0.34	1.53	0.50	0.18	0.53	0.11	0.67	0.14	0.35	0.05	0.34	0.06	0.73
**Sample**	**Zr**	**Nb**	**Mo**	**Ag**	**Cd**	**In**	**Sn**	**Sb**	**Cs**	**Ta**	**W**	**Au**	**Bi**	**Th**	**U**	
*Soda-lime-silica glass with low lime and magnesia*																
**DB-6t**	67.2	2.59	0.16	0.88	0.07	n.d.	1922	3.51	0.09	0.16	3.42	0.01	1.61	1.62	0.46	
**DB-7t**	63.4	2.44	0.15	1.97	0.11	n.d.	1244	3.51	0.13	0.15	6.85	0.03	0.95	1.96	0.71	
**DB-8t**	63.9	2.82	0.12	2.55	0.15	n.d.	6457	12.4	0.10	0.17	2.09	0.48	1.91	1.81	0.55	
**DB-10t**	57.2	2.59	0.11	3.49	0.10	n.d.	1726	9.03	0.07	0.15	0.47	0.06	0.75	1.70	0.56	
**DB-12t**	47.3	1.70	0.10	1.51	0.09	n.d.	4214	4.16	0.07	0.11	10.7	0.04	1.80	1.34	0.33	
**DJ16-12t**	58.7	2.23	0.20	1.58	0.01	n.d.	3052	4.45	0.11	0.15	1.19	0.14	0.93	1.67	0.63	
**DJ17-5t**	78.6	2.23	0.19	1.36	0.25	n.d.	1877	6.89	0.11	0.13	2.62	0.01	1.66	1.69	0.58	
**DB-SM1t**^**§**^	127	3.62	n.a.	n.a.	n.a.	n.a.	n.a.	n.a.	0.12	0.32	n.a.	n.a.	n.a.	2.73	0.74	
**DB-SM2t**^**§**^	131	3.70	n.a.	n.a.	n.a.	n.a.	n.a.	n.a.	0.16	0.35	n.a.	n.a.	n.a.	2.78	0.66	
**DB-2pu**	95.9	2.59	0.69	0.21	0.11	0.46	7145	3.76	0.12	0.16	4.10	0.02	2.30	1.96	0.57	
**DB-3be**	84.6	3.19	0.51	20.2	0.16	n.d.	6859	5.44	0.11	0.20	10.1	7.28	4.15	2.24	0.87	
**DJ16-5t**	49.6	2.56	0.16	3.57	0.18	0.11	190	39.2	0.24	0.17	0.05	0.33	6.50	2.19	0.79	
*Soda-lime-silica glass with high magnesia*																
**DB-9t**	17.5	1.09	0.35	1.73	0.36	n.d.	5894	7.71	0.26	0.11	0.11	0.09	0.95	0.69	0.30	
**DB-11t**	6.61	0.47	0.44	1.60	0.17	n.d.	4456	7.59	0.14	0.03	0.04	0.15	0.76	0.29	0.14	
**DB-4y**	65.8	2.01	1.44	1.01	0.24	n.d.	3969	61.6	0.22	0.14	1.81	0.01	0.78	1.45	0.60	
**DJ16-3b**	65.7	2.05	1.33	0.63	0.09	n.d.	1520	1.37	0.28	0.13	0.62	0.00	0.26	1.46	0.55	
*Soda-lime-silica glass with high alumina*																
**SA11-2g**	76.7	4.19	0.32	2.03	0.05	0.13	3221	28.6	0.22	0.25	0.19	0.03	10.8	1.57	0.41	
*Soda-alumina-silica glass with high boron*																
**DB-5r**	21.0	3.04	0.04	0.07	4522	0.01	0.98	1.30	10.1	0.28	0.26	n.d.	0.05	1.38	0.53	

§Dourou-Boro samples previously analysed.

Concentrations of oxides are given in wt%, concentrations of elements are given in ppm. n.d.: non-detected. n.a.: not available.

### Soda-lime-silica glass with low lime and magnesia

**Fig 6:**
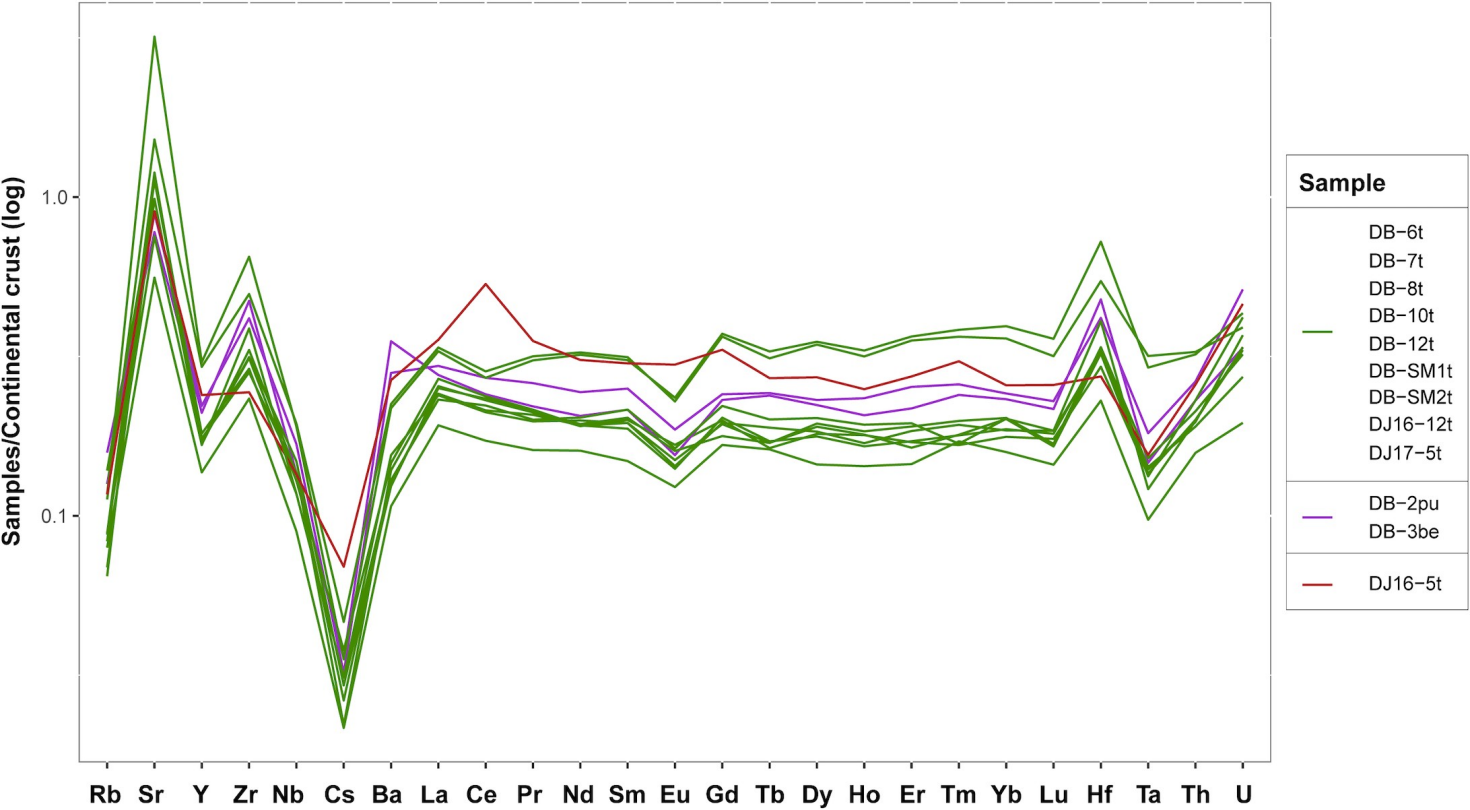
Subdivision of the low lime and magnesia samples based on trace element content.

Twelve monochrome beads found in Dourou-Boro, Mali, and in Djoutoubaya, Senegal, belong to this glass type, namely DB-2pu, DB-3be, DB-6t, DB-7t, DB-8t, DB-10t, DB-12t, DB-SM1t (which shows a slightly higher lime content), DB-SM2t, DJ16-5t, DJ16-12t, and DJ17-5t. Of these beads, ten are turquoise, coloured with copper oxide, whereas two are respectively purple and beige, both probably coloured by manganese and iron. It is important to note that the elemental composition of the latter two glasses is extremely similar despite the colour difference; an analysis using UV-Vis-NIR and Raman spectroscopy would provide more information about the nature of the colouring agents. The glasses in this group show on an average higher levels of Na_2_O and lower concentrations of K_2_O and MgO compared to the other soda-lime-silica glasses from Dourou-Boro and Djoutoubaya, suggesting the use of different plant ashes as a flux (cf. *infra*). Moreover, glass fragment DB-12t shows particularly low concentrations of elements linked to the vitrifier (*e*.*g*. aluminium, titanium, zirconium, rare earth elements) suggesting that it was made with either pure silica sand or quartz pebbles. On the contrary, sample DJ16-5t from Djoutoubaya shows a higher concentration of these elements, probably linked to the use of less pure sand. Three subgroups become evident when considering the trace elements content, the principal group composed of beads DB-6t, DB-7t, DB-8t, DB-10t, DB-12t, DB-SM1t, DB-SM2t, DJ16-12t, and DJ17-5t, the second group composed of beads DB-2pu and DB-3be, and the third group composed by bead DJ16-5t (Fig 6). It must be noted that the trace element concentrations of samples DB-SM1t and DB-SM2t are systematically shifted towards higher values, which could be linked to the different instrumentation and raw data calibration and calculation procedure used for the analysis.

### Soda-lime-silica glass with high magnesia

**Fig 7:**
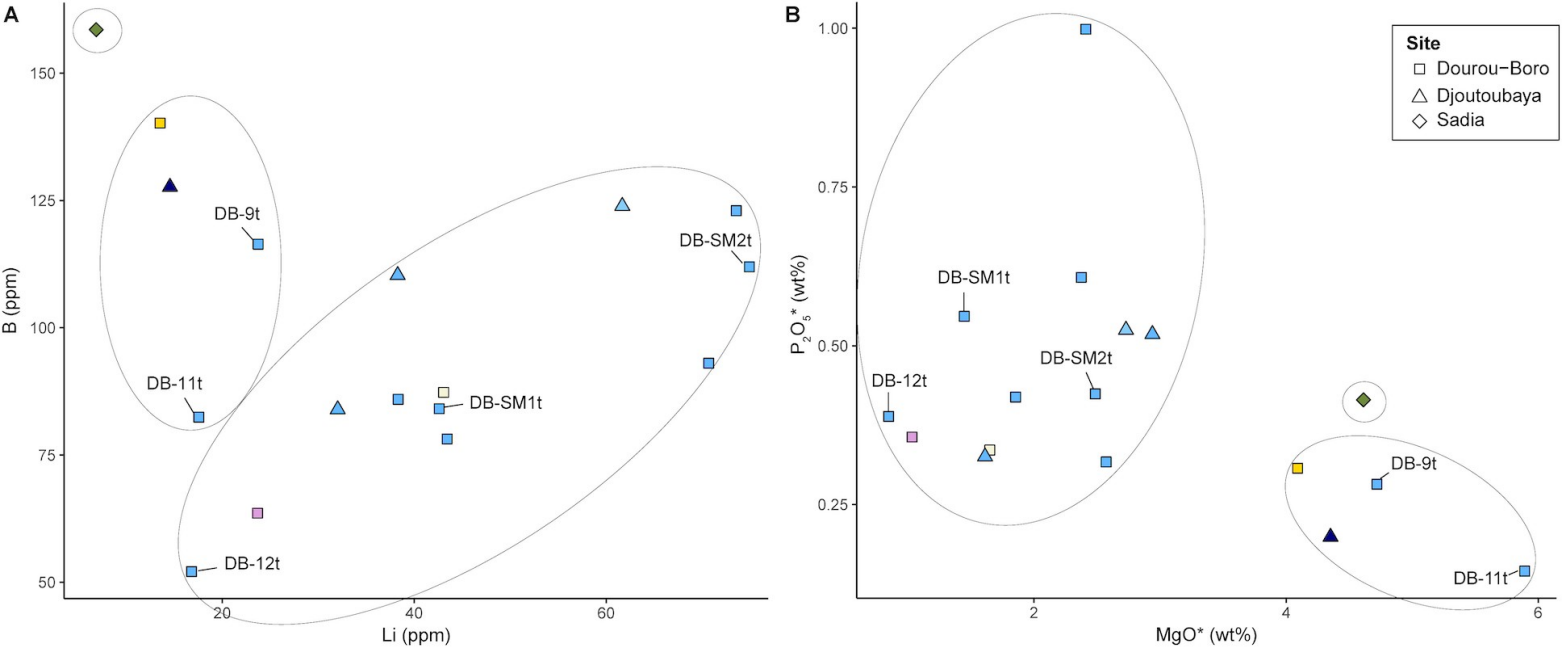
Concentrations of the elements linked to the flux in the analysed glass beads. Boron versus lithium (A) and phosphorus pentoxide versus magnesia (B) suggest different origins for the plant ash used in production.

Three glass beads from Dourou-Boro and one glass bead from Djoutoubaya show this composition, namely DB-4y, DB-9t, DB-11t, and DJ16-3b. All samples contain low levels of Cl and among the lowest levels of P_2_O_5_, as well as a higher concentration of CaO compared to the other soda-lime-silica samples analysed. The concentrations of the elements linked to the flux, such as MgO, K_2_O, P_2_O_5_, Li, and B [3, 39] suggest that the origin of the plant ash is different from the previous samples (Fig 7).

It is interesting to notice that samples DB-9t and DB-11t are typologically consistent with the other turquoise beads from Dourou-Boro (cf. *supra*) but they were produced using different raw materials, the raw glass probably having a different origin. In addition to the different plant ash used, in fact, these two samples show a particularly high level of Sr (547 and 649 ppm, respectively) and a very low concentration of Zr (18 and 7 ppm, respectively), as well as the lowest concentrations of alumina, titanium oxide, and REE, which could be explained by the use of a fairly pure source of silica as vitrifier.

**Fig 8:**
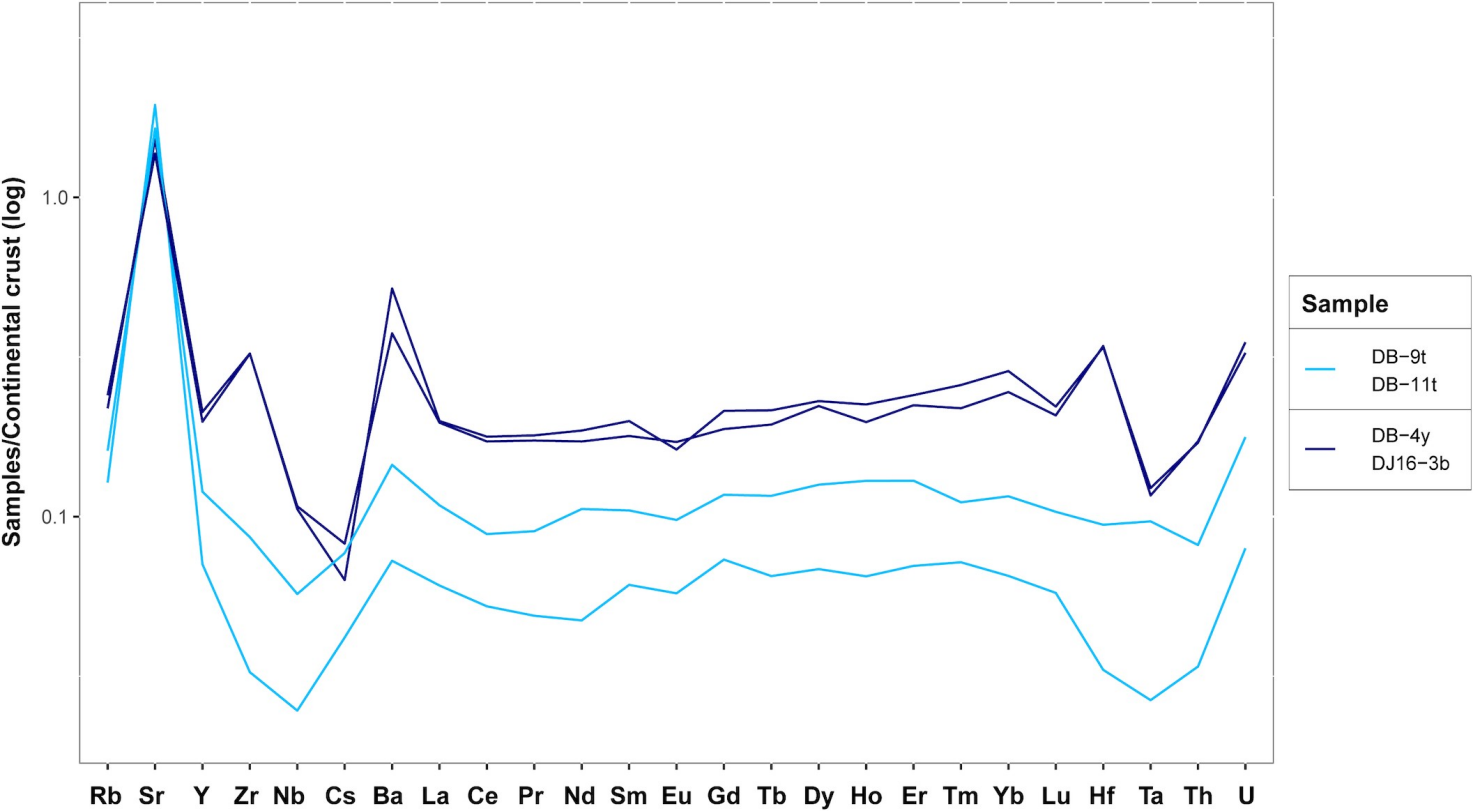
Subdivision of the high magnesia samples based on trace element content.

Considering the trace elements trends, two subgroups can be identified, the first group of DB-9t and DB-11t, and the second of DB-4y and DJ16-3b (Fig 8).

**Fig 9:**
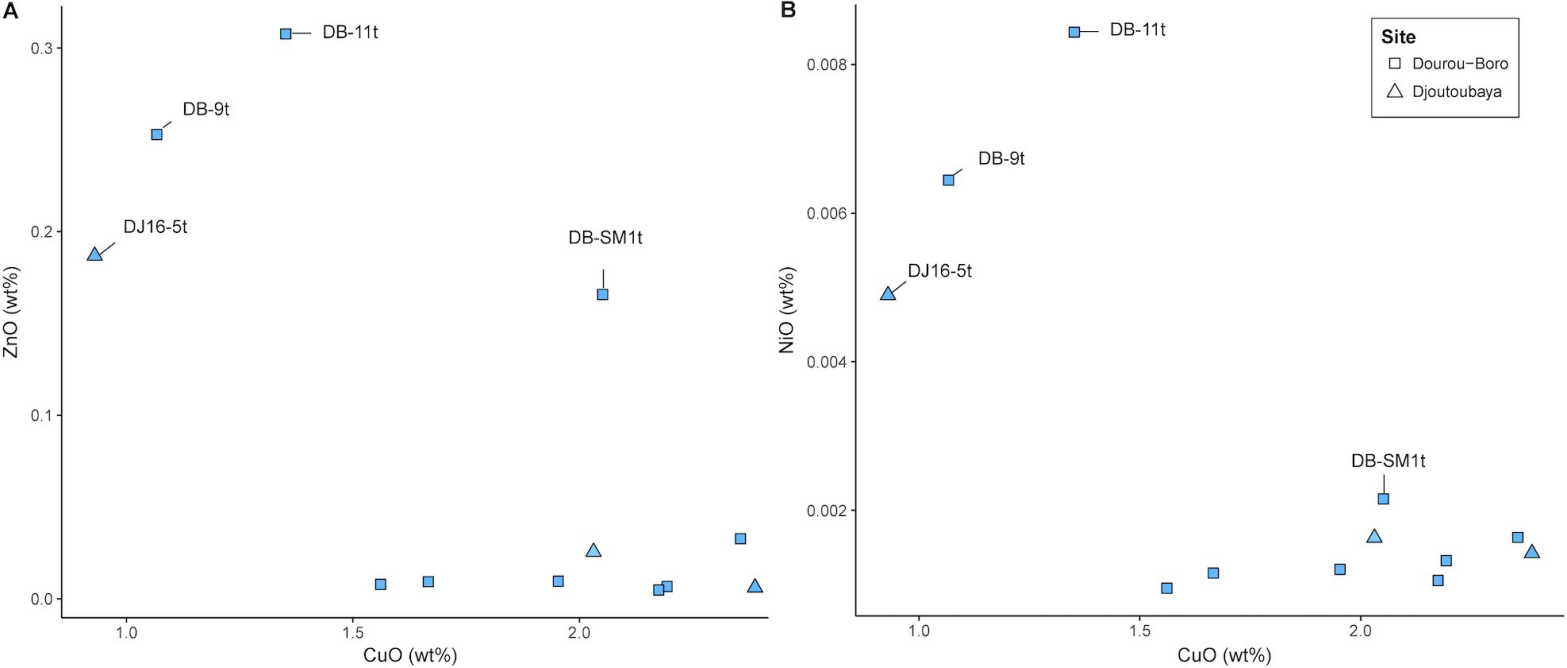
Different origins of the turquoise beads copper colorants. Zinc to copper (A) and nickel to copper (B) show that beads DB-9t, DB-11t, and DJ16-5t have been coloured with the same type of colouring agent, which is different from those used for the other turquoise beads.

As for the colouring agents, the turquoise glasses are coloured by copper oxide, the blue glass by cobalt, whereas the yellow glass is probably coloured by manganese and iron. The chemical equilibrium between Mn^3+^, purple, and Fe^3+^, brown, can in fact produce a yellow tinge in soda-lime-silica glass [40]. The turquoise samples DB-9t and DB-11t contain a considerable amount of zinc and nickel, which are correlated with copper, therefore suggesting the use of the same type of coloring agent, probably brass scraps made from a copper ore containing high levels of nickel. By examining all the turquoise samples from Dourou-Boro and Djoutoubaya, it appears that this correlation is also valid for sample DJ16-5, but not for the other samples, confirming the probable different origin of the beads (Fig 9).

### Soda-lime-silica glass with high alumina

The only sample belonging to this glass type is the green glass bead SA11-2g from Sadia, in Mali. The glass shows high concentrations of Al_2_O_3_ (6.5 wt%), TiO_2_ (0.22 wt%), Ba (368 ppm), and Rb (32 ppm), suggesting the use of an alkali feldspar rich sand as silica source [41]. On the other hand, the fairly high content in MgO (4.4 wt%) and B (159 ppm), and the very low concentration of Li (7 ppm) suggest a different origin of the plant ash used as flux compared to the other analysed beads (Fig 7). Lastly, the green colour of the glass is probably produced by copper oxide.

### Soda-alumina-silica glass with high boron

The red glass fragment DB-5r found in Dourou-Boro, Mali is the only sample presenting this unusual composition. This glass contains very high concentrations of Al_2_O_3_ (14.1 wt%) and B_2_O_3_ (3.5 wt%), as well as low concentrations of MgO, CaO, Cl, and P_2_O_5_. The red colour of the glass is produced by the complex formed by Cd (4522 ppm) and Se (1814 ppm).

## Discussion

### Hypothesis on the glass provenance

In order to determine the possible origin of the samples, the glassmaking centres active during the second half of the 1^st^ and the first half of the 2^nd^ millennium CE must be taken into account. In view of the composition of the above discussed samples, it is possible to confirm that the red glass fragment from Dourou-Boro falls outside this chronology, since it is a modern production: soda-alumina-silica glass with high boron was in fact developed after the 19^th^ century CE [42] and the cadmium-selenium colorant was not used before the end of the 19^th^ century or after the turn of the 20^th^ century but was commonly used in decorative pressed glass by the 1920s or 1930s [43]. This glass fragment is therefore the result of site contamination, probably due to grave looting [1].

**Fig 10:**
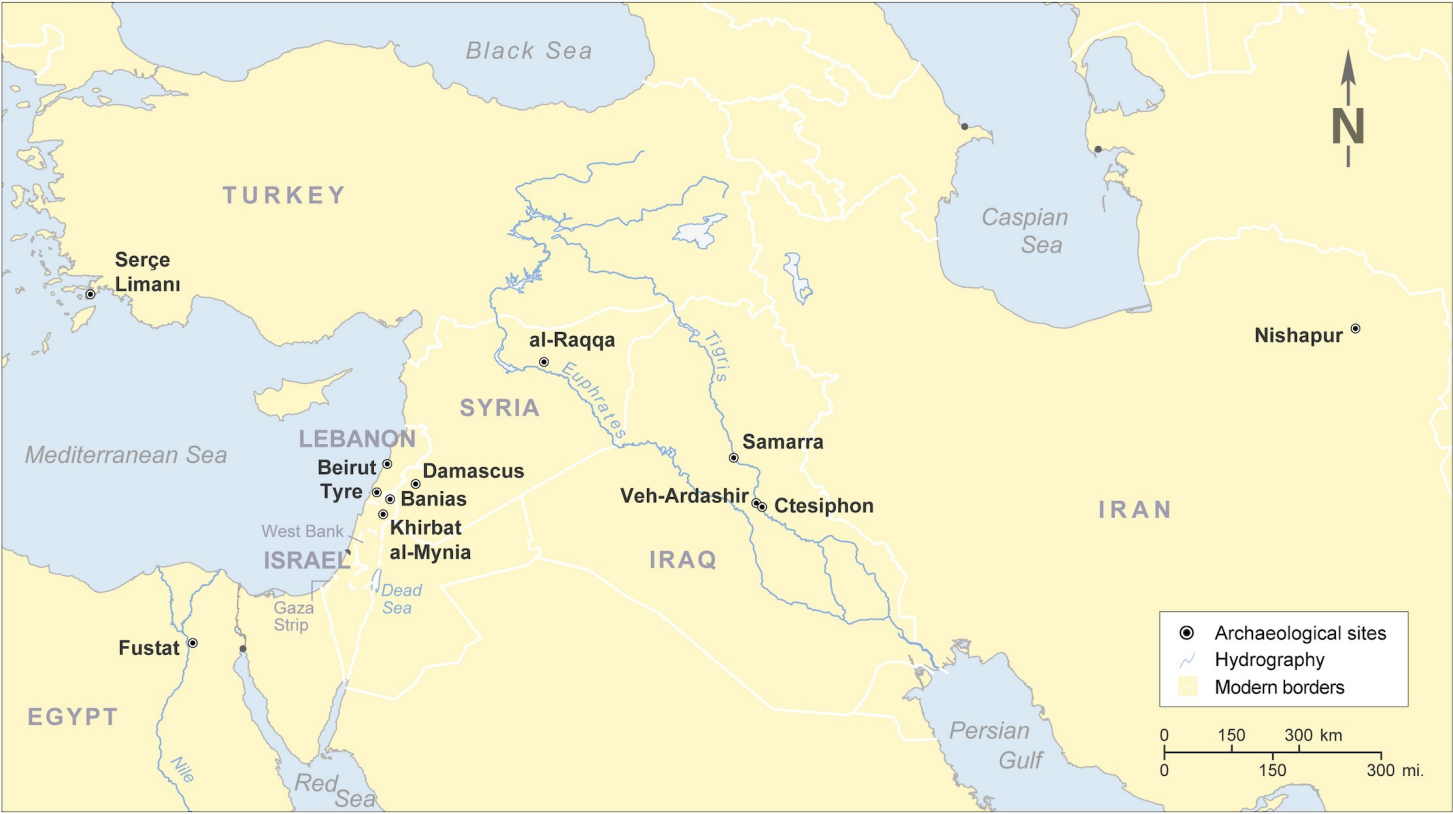
Location of the archaeological sites where the plant-ash glasses used as reference were found (map: Courtesy of CIA’s *The World Factbook* 2020, modified).

As for the other samples from Dourou-Boro and Djoutoubaya, various collections of archaeological plant-ash glasses found in several sites located in the Mediterranean basin and the Middle East were used as reference material (Fig 10).

**Fig 11:**
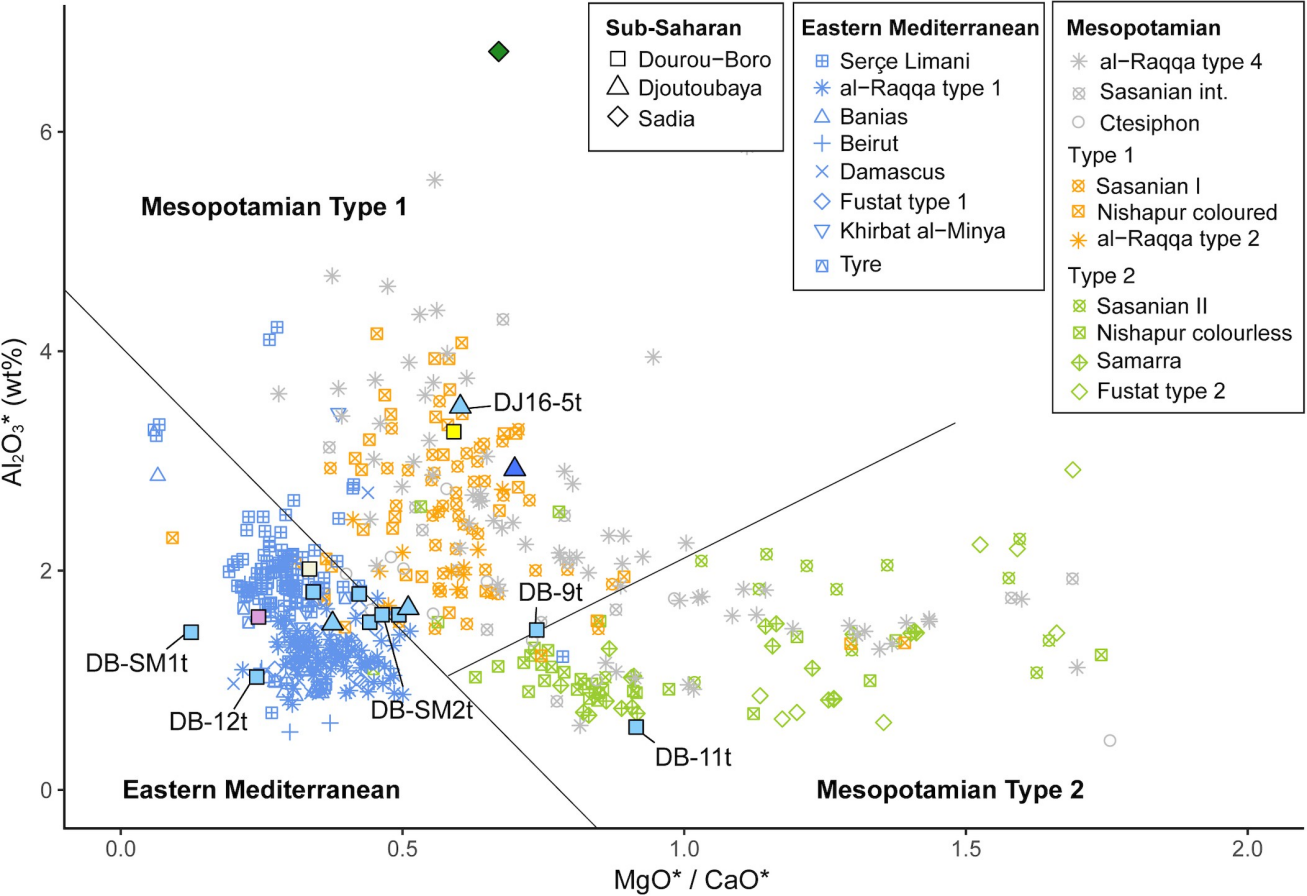
Dourou-Boro, Djoutoubaya, and Sadia glasses compared to contemporary glass assemblages. Alumina versus magnesia to lime ratios of published data of glasses from Levantine and Mesopotamian contemporary sites, grouped after Phelps [49, 50].

As mentioned above, plant ash flux was reintroduced instead of natron in the near-eastern productions west of the Euphrates River in the 8^th^ century CE [3, 44, 45]. On the other hand, in Mesopotamia, Iran, and Central Asia, plant-ash glass has probably been in constant use since the beginning of the 1^st^ millennium CE [3, 39, 46–48]. The glasses from Tyre, Banias, al-Raqqa, Samarra, Veh-Ardashir, and Nishapur have been grouped by Phelps into two main compositional groups based on their alumina, lime, and magnesia content, namely the Eastern Mediterranean group (Syro-Palestinian and Egyptian glass) and the Mesopotamian group (Iranian and Iraqi glass), the latter being further divided into two types [49, 50]. The glass assemblages of several other sites, namely Serçe Limani, Beirut, Damascus, and Fustat [37], as well as Khirbat al-Minya and Ctesiphon [51] have been added to this initial clustering. Fig 11 shows how all the samples from sites located in the region west of the Euphrates River fall into the Eastern Mediterranean group, except for the type 2 glass from Fustat, which is chemically compatible with the Mesopotamian Type 2 glass. At al-Raqqa, three main plant-ash compositions were found [3, 52]; type 1 (and sub-type 1) having a typical Eastern Mediterranean composition, and type 2 and 4 falling into Phelps’ Mesopotamian group. Finally, Sasanian I and II glasses from Veh Ardashir [39, 47], as well as coloured and colourless glass from Nishapur [53] show a Mesopotamian composition (Type 1 and Type 2, respectively for each site).

If the sub-Saharan African samples are compared with the reference materials (Fig 11), we can see that the soda-lime-silica glasses with low lime and magnesia from Dourou-Boro and Djoutoubaya show a similar composition to the Eastern Mediterranean productions, except for sample DJ16-5t that has a different trace element pattern (Fig 6); moreover, the soda-lime-silica glasses with a high magnesia content from Dourou-Boro and Djoutoubaya are comparable to the Mesopotamian productions, being separated into Phelps’ Types 1 and 2 according to the subgroups identified in the trace element patterns (Fig 8). The soda-lime-silica glass with high alumina content from Sadia, on the other hand, does not show the typical composition of Islamic productions (cf. *infra*).

**Fig 12:**
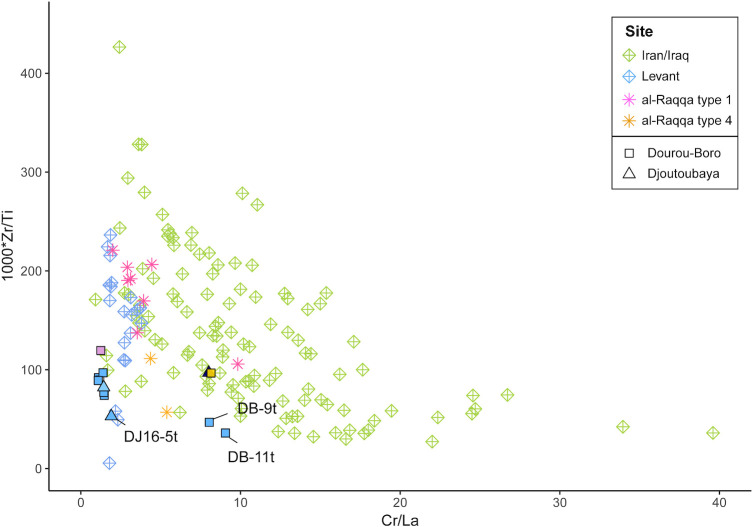
Trace elements composition of Dourou-Boro and Djoutoubaya glasses compared to contemporary glasses. Zirconium to titanium versus chromium to lanthanum ratios of glasses from Dourou-Boro and Djoutoubaya compared to Levantine and Mesopotamian glasses showing the different sources of raw materials.

These attributions are confirmed by the trace elements composition of the samples, as shown by the Cr to La and Zr to Ti ratios (Fig 12). The chromium to lanthanum ratio, in particular, tends to increase in value and variability moving from west (the Levantine coast) to east (the Middle East), due to the geochemistry of the mountain chains and river valleys [51]. Fig 12 shows that our high magnesia glasses are compatible with the samples from Nishapur, Samarra, and Ctesiphon (Iran/Iraq), whereas low lime and magnesia glasses are comparable with those from Beirut, Damascus, and Khirbat al-Minya (Levant).

**Fig 13:**
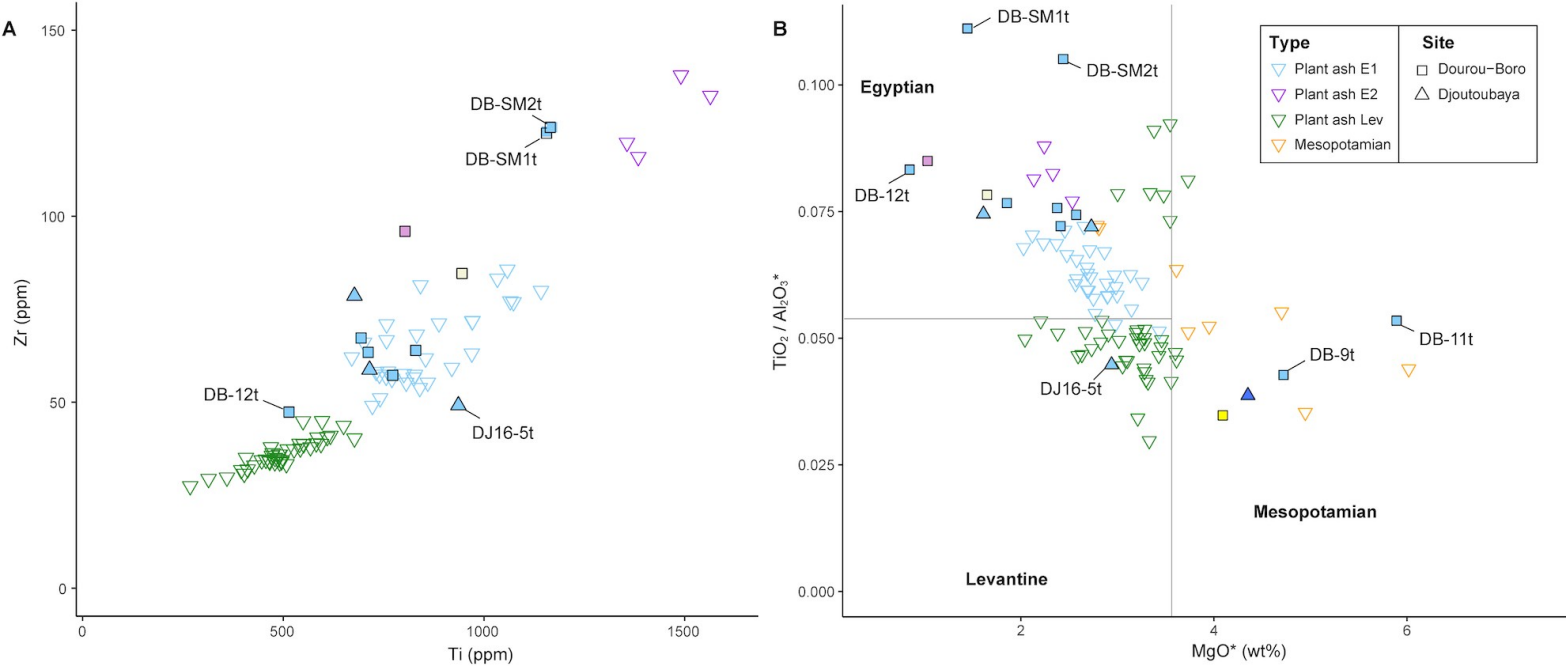
Dourou-Boro and Djoutoubaya glasses compared to Egyptian, Levantine, and Mesopotamian glasses. Titanium oxide to alumina ratio versus magnesia (A) and zirconium versus titanium (B) confirm the probable different origin of the glasses from Dourou-Boro and Djoutoubaya.

The composition of Islamic glass weights and stamps from Egypt dated between 700 and 1020 CE analysed by Schibille *et al*. was taken into account in order to better understand the origin of the Levantine glass [54]. In this study, they were able to distinguish between a strictly Levantine origin (Lev) and an Egyptian origin (E1 and E2) of the glasses based on the MgO, Ti, and Zr contents, as well as the TiO_2_ to Al_2_O_3_ ratio. Fig 13 shows how the composition of the low lime and magnesia beads from Dourou-Boro and Djoutoubaya is more similar to the Egyptian weights and stamps composition rather than the Levantines ones. Caution should be however applied to this interpretation because of the very different nature of artefacts compared, for which different choices of raw materials and manufacture criteria might have been made.

Finally, it needs to be mentioned that the possibility of some beads being made by mixing glass from different sources cannot be ruled out, especially considering their lead content, as well as the variability of the concentration of the trace elements linked to the colorants.

**Fig 14:**
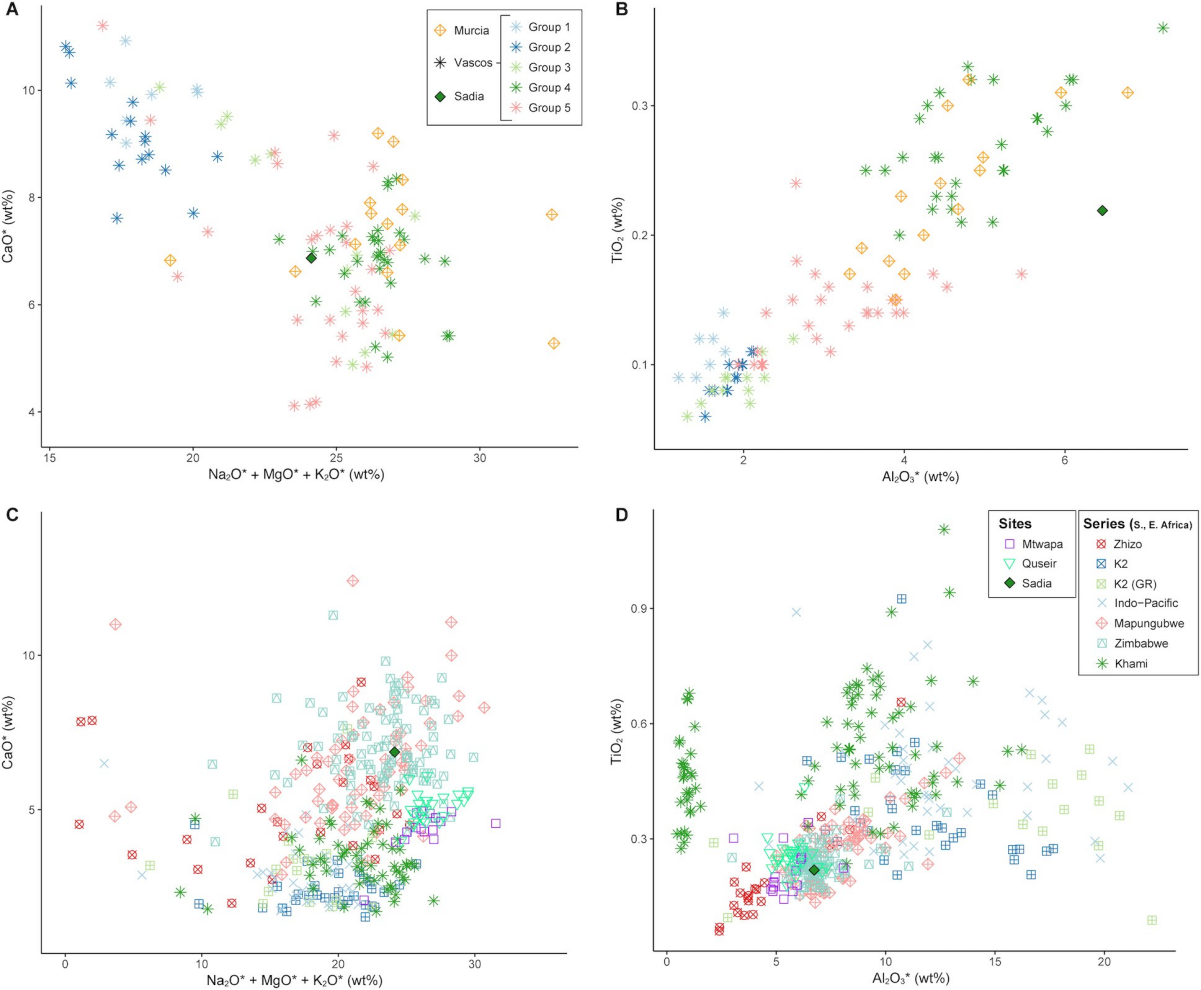
The Sadia glass bead compared to contemporary high alumina glasses. Lime versus the sum of soda, magnesia and potash (A, C) and titanium oxide versus alumina (B, D) of high alumina glass from Sadia compared to contemporary glasses from the Iberian Peninsula (A, B) and from eastern and southern Africa (C, D).

As mentioned above, the Sadia bead shows a composition that is fairly different from “classic” Islamic glass composition because of its high Al_2_O_3_ content. High alumina glass is actually more typical of south and southeast Asia, where it was produced principally with a mineral flux [55, 56]. However, high alumina plant ash glass having a composition similar to the Sadia bead, the so-called *vNCA* glass in [55] (pp. 130–131, 137–138), has been found in south Asian contexts dated between the 1^st^ century BCE and the 1^st^ century CE; these were not taken into account as reference material in view of the chronological discrepancy. On the other hand, high alumina glass, glass waste, and beads were found in contexts within the chronological range of the Sadia bead in the Iberian Peninsula [57, 58], and in eastern and southern Africa [59–61]; however no primary production site has been yet found for most of these materials. Fig 14 shows that, in terms of its main composition, SA11-2g is similar to the glasses from Murcia (12^th^ century CE) and Ciudad de Vascos (groups 4 and 5, 10^th^-11^th^ century CE) in Spain (Fig 14A and 14B), to the Mapungubwe Oblate bead series (13^th^-14^th^ century CE) and Zimbabwe bead series (14^th^ -15^th^ century CE) from southern Africa, as well as to the glasses from Mtwapa (10^th^ -18^th^ century CE) in Kenya and from Quseir el-Qadim (13^th^ -14^th^ century CE) in Egypt (Fig 14C and 14D).

**Fig 15:**
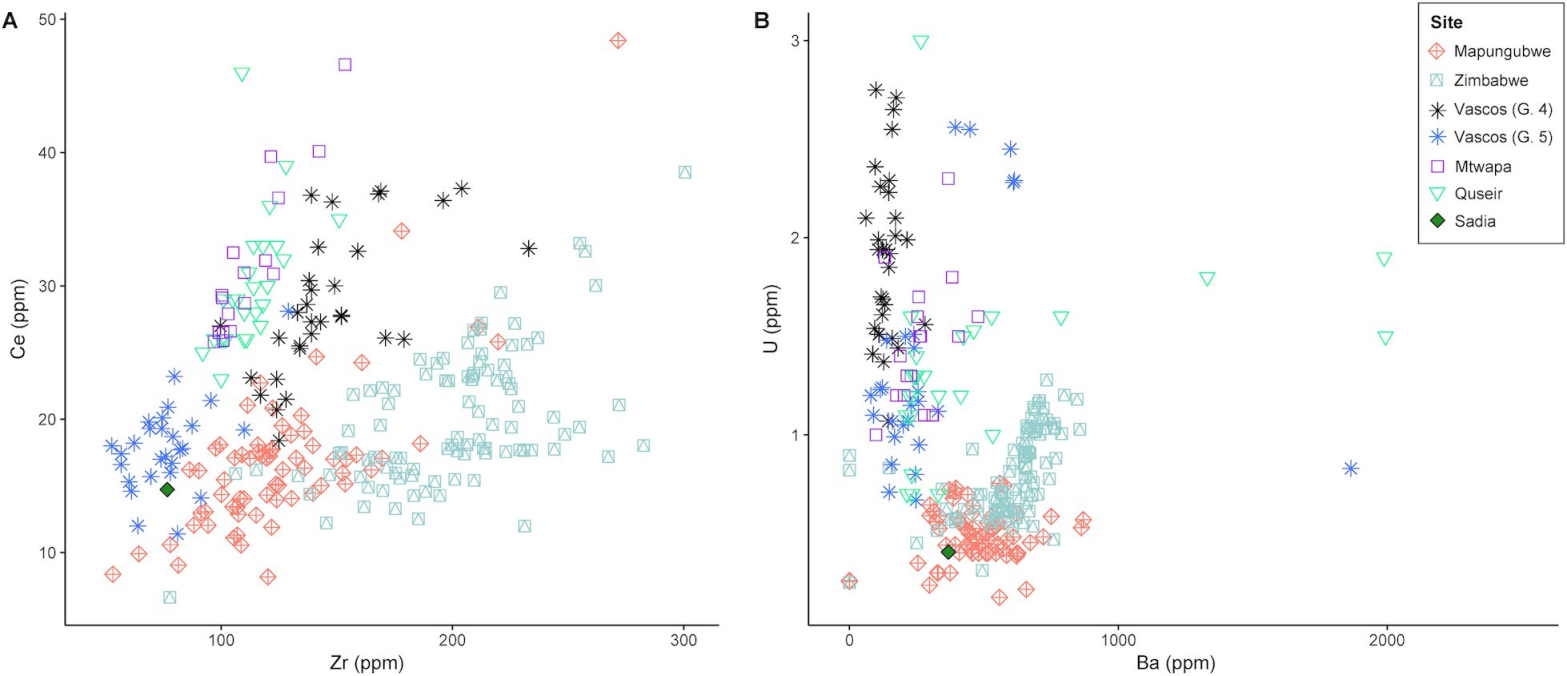
Trace element composition of the Sadia bead compared to contemporary high alumina glasses. Cerium versus zirconium (A) and uranium versus barium (B) of the Sadia bead compared to Iberian, southern, and eastern African glasses show compatibility with group 5 glass from Ciudad de Vascos and with South African Mapungubwe Oblate.

Considering the Zr, Ce, U, and B composition, it is possible to further refine the attribution, leaning towards the Mapungubwe Oblate bead series on one side, and group 5 glass from Ciudad de Vascos on the other (Fig 15).

From a typological point of view, the Mapungubwe Oblate beads have a very specific morphology, which is quite different from the Sadia bead. However, the shape of SA11-2g suggests that its ends were ground flat after production. Considering that the secondary modification of imported glass beads was a fairly common practice in West Africa, it cannot be excluded, based on its morphology, that Sadia bead could be a Mapungubwe Oblate bead modified afterward (Marilee Wood, pers. comm.). Nevertheless, considering the unknown origin of the glasses taken into account for comparison, it is impossible to make assumptions on the actual provenance of the Sadia bead, although the exchange with northern Africa through the Sahara seems more plausible on account of geographical considerations.

### Comparison with other West African bead assemblages

As mentioned in the introduction, many glass beads were found in several West African archaeological sites dated between the 8^th^ and the 15^th^ centuries CE. Table 4 lists the assemblages taken into account for comparison, indicating the site and the chronology of the find contexts of the beads, as well as the type of glasses considered for comparison, namely plant-ash soda-lime glass with medium/high magnesia content (*v-Na-Ca*), plant-ash soda-lime glass with low magnesia content (*v-Na-Ca low Mg*), and plant-ash soda-lime glass with high alumina content (*v-Na-Ca high Al*). The bibliographic references for the chemical compositions are also indicated.

**Table 4:** West African glass bead assemblages considered for comparison.

Site	Chronology	Glass types			References
		v-Na-Ca	v-Na-Ca low Mg	v-Na-Ca high Al	
**Jenne-Jeno (Mali)**	4^th^-15^th^ c. CE	X			[53]
**Gao (Mali)**	7^th^-13^th^ c. CE	X			[62]
**Kissi (Burkina Faso)**	end 1^st^ mill. CE	X	X		[63]
**Essouk (Mali)**	10^th^-14^th^ c. CE	X	X	X	[64]
**Igbo-Ukwu (Nigeria)**	7^th^-13^th^ c. CE	X		X	[26]
**Al-Basra (Morocco)**	9^th^-11^th^ c. CE	X		X	[65]

*v-Na-Ca*: plant-ash soda-lime glass with medium/high magnesia; *v-Na-Ca low Mg*: plant-ash soda-lime glass with low magnesia; *v-Na-Ca high Al*: plant-ash soda-lime glass with high alumina.

**Fig 16:**
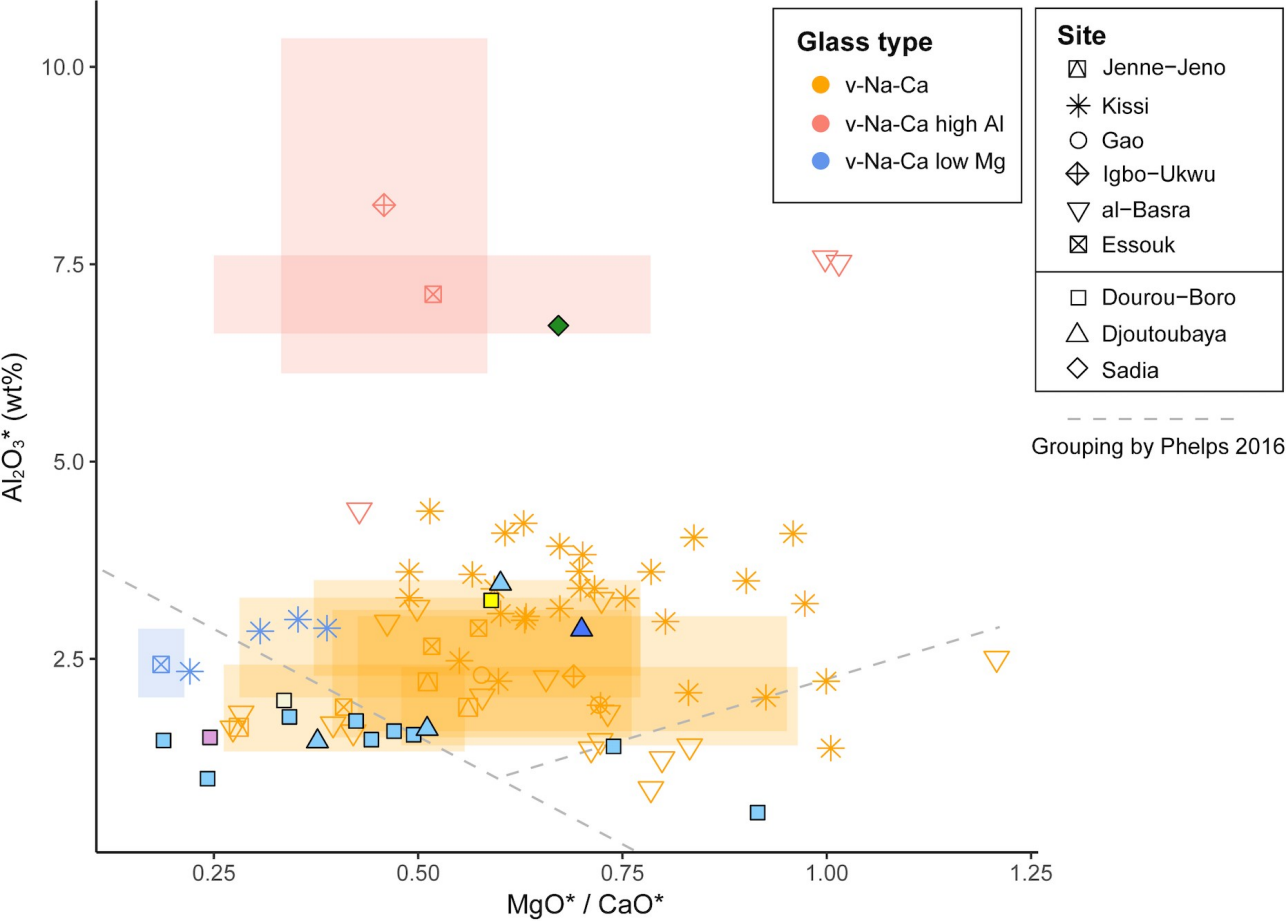
Comparison between contemporary glass bead assemblages from West Africa. Alumina versus magnesia to lime ratios of published compositions of glass beads from West Africa, grouped after Phelps [49, 50].

By plotting the alumina content versus the magnesia to lime ratio and considering the Eastern Mediterranean and Mesopotamian grouping by Phelps [49, 50], it is possible to see that the majority of the chemically analysed beads found in West Africa have a soda-lime with high magnesia composition (Fig 16), corresponding to the Phelps Mesopotamian Type 1 group and al-Raqqa type 4 glass (cf. Fig 11).

High magnesia glasses of probable Middle Eastern production from Dourou-Boro and Djoutoubaya find thus correspondences in the Jenne-Jeno, Kissi, Gao, Igbo-Ukwu, and al-Barsa assemblages, as well as in the Essouk assemblage that was found in contexts dated between the 12^th^-13^th^ and the 14^th^ century CE. However, the low alumina beads DB-9t and DB-11t seem to have a composition less common in West Africa, which is otherwise found in al-Basra, Morocco. On the other hand, the low lime and magnesia glasses from Dourou-Boro and Djoutoubaya, previously identified as probable Levantine productions, are similar to the *v-Na-Ca* beads from Kissi and Essouk (10^th^-11^th^ to 12^th^ century CE), having however a lower alumina content. Finally, beads with high alumina content similar to the Sadia bead have been found in Essouk, Igbo-Ukwu, and al-Barsa.

It is noteworthy that no high lime high alumina glass bead of Nigerian primary production (cf. *supra*) are present in this collection, in spite of the fact that beads with this specific composition were found in many contemporary West African archaeological sites. This could be explained by the structure of the sub-Saharan trade routes at the time, which were mainly developed on a north-south axis, linking the cities located along the Niger River and in southern Mauretania to the Mediterranean basin and the Levant, as opposed to an east-west axis through the savannah regions.

### Integration of the peripheral sites in the long-distance trade

The three archaeological sites are located in rural areas of the Niger bend (Dourou-Boro and Sadia) and of the Falémé river valley (Djoutoubaya). While the first two are situated in a region rich in archaeological sites that have imported glass beads assemblages, the last one is far from the urban centres involved in the long-distance trade. Djoutoubaya is actually located in one of the areas of gold production that was active between late 1^st^ and early 2^nd^ millennia CE [20, 66], a region where very few glass beads have been found for the period in question, and for which no chemical analysis has been performed. This is also true in general for glass beads assemblages found outside the major sites involved in the trans-Saharan trade, which is the usual focus for research. The chemical characterization of the glass beads from Dourou-Boro, Sadia, and Djoutoubaya provides therefore new insights on the ramification of the long-distance trade on a local level and new compositional data for the growing literature of glass analysis in West Africa. The comparison of these glass beads compositions with those from assemblages found in other West African sites shows how the exchange of these imported materials was not only for the elites at the main centres but it continued, on a smaller scale, to non-urbanised areas. By taking into account the organisation of the trans-Saharan trade routes between the 7^th^ and the 15^th^ centuries CE, a scenario can be constructed in which the three sites were probably connected to different circuits. Based merely on a geographical evaluation, it seems in fact more probable that Dourou-Boro and Sadia were linked to the trade centres of Essouk, Gao, and Timbuktu, whereas Doutoubaya was connected to the caravan towns of Tegdaoust and Koumbi Saleh (Fig 1). Interestingly enough, there seems to be no preferential distribution of specific bead types through different trade routes, as beads with the same composition are found equally in both regions. The chronology of the sites and the area of production of the glass do not appear to be factors either. It is however important to point out that more analyses of glass beads from other contemporary minor sites are needed in order to corroborate these hypotheses.

## Conclusion

A total of 16 glass beads found in West African archaeological contexts dated between the 7^th^ and the 13^th^ centuries CE in Dourou-Boro and Sadia, Mali, and in Djoutoubaya, Senegal, were analysed by LA-ICP-MS. Two beads from Dourou-Boro previously analysed in another laboratory were included in the study as well. Four chemical groups were identified based on the main composition of the glasses, namely soda-lime silica glass with low lime and magnesia (12 samples), soda-lime silica glass with high magnesia (4 samples), soda-lime silica glass with high alumina (1 sample), and soda-alumina-silica glass with high boron (1 sample). The glass of the first three groups were produced using sands with differing levels of purity as vitrifier and various plant ash types as flux, whereas the glass fragment from the last group is a modern production, introduced on site probably by grave looters. Each main group was further divided into subgroups based on the trace element composition, indicating that different raw materials were probably used for production. This is also true for the typologically identical turquoise beads from Dourou-Boro, for which at least two sources of raw materials have been identified. The comparison of the compositions with the chemical data of contemporary glasses suggests an Eastern Mediterranean production for the low lime and magnesia glasses, pointing specifically towards Egypt and the Levantine coast, with similarities as well with the type 1 glasses from the primary production site of al-Raqqa, in Syria; on the other hand, the origin of the high magnesia glasses is probably the Mesopotamian region, east of the Euphrates River, with at least two different sources of raw materials; finally, the provenance of the high alumina glass bead is unclear, but its composition can be related to some Iberian glasses from Ciudad the Vascos, and beads of the Mapungubwe Oblate series from South Africa, both of unknown origin.

The analysed glass beads were therefore imported in West Africa through the trans-Saharan trade routes during the early phase of the Islamic expansion in North Africa, and in the context of the development of the first states in Sub-Saharan Africa, such as the Ghana and the Gao kingdoms. This is confirmed by the fact that the composition of the beads is in line with the compositions of glass beads found in several other West African sites, and in particular in the major urban centres involved in the trans-Saharan trade. Two different parts of the commercial network linking both edges of the Sahara were probably used to reach Djoutoubaya on one side, and Dourou-Boro and Sadia on the other, according to their geographical proximity to different urban trade centres, respectively Tegdaoust and Koumbi Saleh on one side, and Essouk, Gao, and Timbuktu on the other. The three sites analysed here were linked to these trade routes in spite of the remoteness of their location, attesting the distribution of this kind of small imported luxury goods into the rural areas of West Africa through regional and local exchange networks reaching villages. Lastly, the Djoutoubaya discoveries bring new insights on the integration of the gold production areas into this multi-scalar trade network. Finally, it questions the role of these peripheral sites producing important resources, like gold or ivory, within the political and economic organization of the first Sub-Saharan states.
